# Anti-inflammatory and antimicrobial efficacy of coconut oil for periodontal pathogens: a triple-blind randomized clinical trial

**DOI:** 10.1007/s00784-025-06267-8

**Published:** 2025-03-14

**Authors:** Simón Pardiñas López, Mónica E. García-Caro, Juan A. Vallejo, Pablo Aja-Macaya, Kelly Conde-Pérez, Paula Nión-Cabeza, Ismael Khouly, Germán Bou, Ana Isabel Rodríguez Cendal, Silvia Díaz-Prado, Margarita Poza

**Affiliations:** 1 Periodontology and Oral Surgery, Clínica Médico Dental Pardiñas, Real 66, 3, A Coruña, 15003 Spain; 2 Grupo de Terapia Celular y Medicina Regenerativa, Instituto de Investigación Biomédica de A Coruña (INIBIC), Servizo Galego de Saúde (SERGAS), Complexo Hospitalario Universitario de A Coruña (CHUAC), A Coruña, 15003 Spain; 3 Grupo de Terapia Celular y Medicina Regenerativa, Departamento de Fisioterapia, Medicina y Ciencias Biomédicas, Facultad de Ciencias de la Salud-Centro Interdisciplinar de Química y Biología (CICA), Universidade da Coruña, A Coruña, 15701 Spain; 4 Grupo de Investigación en Microbiología, Servicio de Microbiología, Instituto de Investigación Biomédica de A Coruña (INIBIC)- Hospital Universitario de A Coruña (CHUAC)-Universidade da Coruña (UDC)-CIBER de Enfermedades Infecciosas (CIBERINFEC, ISCIII), Hospital Universitario, Coruña, 15006 A Spain; 5 Grupo Microbioma y Salud, Facultad de Ciencias- Centro Interdisciplinar de Química y Biología (CICA), Universidade da Coruña, A Coruña, 15071 Spain; 6 Department of Oral and Maxillofacial Surgery, College of Dentistry, New York University, New York, NY 10010 USA; 7 Multidisciplinary Implant and Aesthetic Miami Institute (M.I.A.M.I.), Miami, FL 33137 USA

**Keywords:** Periodontitis, Oral Microbiome, Coconut oil, 16S rRNA sequencing, Inflammation, Chlorhexidine

## Abstract

**Objectives:**

To evaluate the effect of coconut oil on the oral bacteriome and inflammatory response in patients with periodontitis by integrating next-generation sequencing analyses of pathogenic bacterial shifts and quantification of inflammatory markers, thereby assessing its potential as a natural adjunct to standard nonsurgical periodontal therapy.

**Materials and methods:**

A triple-blind clinical trial was conducted with 30 participants diagnosed with periodontitis, randomized into 3 groups: (1) coconut oil, (2) chlorhexidine and (3) placebo. Saliva and gingival crevicular fluid (GCF) samples were collected before treatment, one month after treatment, and one month post-non-surgical periodontal therapy. Bacterial DNA was extracted, and the V3-V4 region of the 16 S rRNA gene was PCR-amplified and sequenced using Illumina MiSeq technologies. Inflammatory biomarkers, including Interleukin-6 (IL-6) and tumor necrosis factor-alpha (TNF-α), were quantified from GCF samples.

**Results:**

Coconut oil treatment significantly reduced pathogenic bacterial families such as *Spirochaetaceae* and *Tannerellaceae* while promoting beneficial bacteria such as Streptococcaceae. At the genus and species levels, coconut oil reduced pathogens such as *Tannerella forsythia* and *Treponema denticola* along with increase in beneficial bacteria such as *Streptococcus*. The subgingival microbial dysbiosis index improved significantly in both coconut oil and chlorhexidine groups. Furthermore, the coconut oil demonstrated a reduction in IL-6 and TNF-α levels, indicating decreased local inflammation.

**Conclusions:**

Coconut oil treatment significantly modulated the oral microbiome and reduced inflammatory markers in patients with periodontitis, suggesting its potential as a natural and effective adjunct in periodontal therapy.

**Clinical relevance:**

This study highlights coconut oil’s potential as a natural adjunct in periodontal therapy, effectively reducing pathogenic bacteria and inflammatory markers (IL-6, TNF-α). It offers a safe alternative to chlorhexidine, promoting microbiome balance and improved periodontal health.

**Supplementary Information:**

The online version contains supplementary material available at 10.1007/s00784-025-06267-8.

## Graphical Abstract

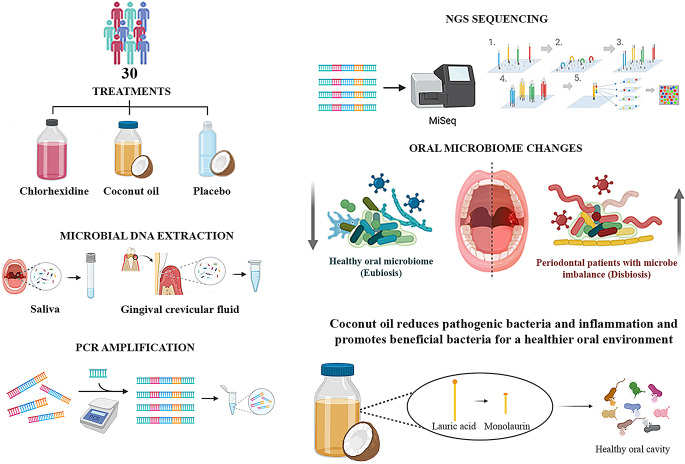

## Introduction

Periodontitis, a chronic inflammatory disease resulting from microbiome dysbiosis that affects the supporting structures of the teeth is the sixth most prevalent chronic disease worldwide [1] and remains a significant global oral health issue [2–4]. 

Its pathophysiology involves key molecular pathways that activate host-derived proteinases, causing the loss of marginal periodontal ligament fibers, the downward migration of the junctional epithelium, and the apical spread of bacterial biofilm along the root surface [2]. 

In the mouth, bacteria aggregate into biofilms within distinct niches, each supporting specific microbial populations shaped by unique environmental conditions. This dynamic nature of the oral microbiome makes defining a general composition difficult [5–8]. 

In healthy conditions, the bacterial species that compose the biofilm are primarily aerobic organisms, recognized by the host’s immune system as harmless. They protect the host from pathogens and maintain a balance that allows symbiotic relationships, known as microbial homeostasis or eubiosis [7, 8].

However, poor oral hygiene and factors such as a poor diet or smoking disrupt this balance, leading to an overproliferation of pathogenic species known as dysbiosis that promotes the development of oral diseases [7, 8]. This oral microbiome’s complexity, influenced by individual health, limits classical bacterial culture methods, especially for uncultivable bacteria in dysbiotic states like periodontitis [9] Next-generation sequencing (NGS), particularly 16 S rRNA metabarcoding, has advanced microbiome research by identifying uncultivable microorganisms and reducing costs [10, 11]. This technique uses 16 S rRNA gene fragments to distinguish bacteria associated with health or dysbiosis and assess treatment effects.

Furthermore, inflammation is a hallmark of periodontitis, with several pro-inflammatory cytokines playing a crucial role in their pathogenesis. Notably, IL-6 and TNF-α are two key cytokines that significantly contribute to the inflammatory process associated with periodontitis. IL-6 is involved in the regulation of immune responses and acts as a mediator of inflammation by promoting the differentiation of B cells and the activation of T cells. Elevated levels of IL-6 in periodontal tissues are strongly associated with the progression of periodontitis and the destruction of the periodontal attachment [12]. 

TNF-α, on the other hand, plays a pivotal role in orchestrating the local inflammatory response and the breakdown of connective tissue and bone. By stimulating the production of matrix metalloproteinases, TNF-α facilitates the degradation of extracellular matrix components, contributing to the apical migration of the junctional epithelium and alveolar bone loss [13]. These cytokines also enhance the expression of other pro-inflammatory mediators, perpetuating a cycle of inflammation and tissue destruction that characterizes periodontitis [14]. 

Adjuvant rinses with antiseptic properties, including chlorhexidine (CHX), essential oils, and cetylpyridinium chloride, have been suggested as effective methods to control the progression of periodontitis by targeting dental plaque and reducing inflammation [15].

Among these, CHX is widely used for managing oral pathologies due to its broad-spectrum antimicrobial activity [15] However, prolonged CHX use is linked to side effects such as tooth and tissue staining, taste alteration, burning sensations, and type 1 hypersensitivity reactions [5].

On the other hand, natural options like coconut oil (CO) have been gaining more attention for their potential advantages in maintaining oral hygiene. CO is largely composed of medium-chain fatty acids such as lauric acid and capric acid that have antimicrobial properties against a wide range of microorganisms. In the case of Gram-negative bacteria, the amphipathic nature of these compounds allows them to penetrate the bacterial membrane and form micelles that disrupt the membrane, leading to increased permeability, leakage of cell contents, and ultimately cell death [16, 17]. In Gram-positive bacteria, lauric acid can interact with the enzyme that forms peptidoglycan bonds, leading to cell lysis [18, 19]thereby reducing dental plaque and inflammation [18, 20–22].

Beyond its direct antimicrobial activities, lauric acid can be converted in the body to monolaurin (glycerol monolaurate), a compound similarly reported to have strong inhibitory effects on various pathogenic organisms [17, 20, 23]. 

CO has been found to be a cost-effective and easy-to-obtain option for maintaining good dental hygiene [24]. While in vitro studies show CO impacts biofilms, sequencing data on its oral health effects remain limited [25].

To our knowledge, this study is the first triple-blind, randomized controlled clinical trial evaluating the oral microbiological and inflammatory response of CO rinse as adjunct periodontal therapy.

## Materials and methods

### Study design

This single-center, triple-blinded randomized controlled trial evaluated the clinical efficacy of CO, 0.12% CHX and placebo mouthwashes as adjunctive therapies in periodontal treatment. The study adhered to the CONSORT 2010 guidelines for reporting randomized clinical trials [26] as well as the recommendations of the European Federation of Periodontology [27] and Cochrane’s risk of bias tools [28].

### Ethical approval

The study protocol, approved by the Comité de Ética de la Investigación con Medicamentos de Galicia (CEIm-G) under protocol number 2017/247, was registered at ClinicalTrials.gov (NCT06049589). Conducted in accordance with the Declaration of Helsinki and Good Clinical Practice guidelines, all participants provided written informed consent prior to enrollment. The study was conducted at Fundación Clínica Pardiñas, INIBIC, and Hospital Universitario de A Coruña between November 2022 and December 2023, prioritizing patient safety, validity, and reproducibility.

### Eligibility criteria for participants

#### Inclusion criteria

Human patient with over 18 years diagnosed with periodontal disease stages II and III (grades B and C) based on the 2017 World Workshop on the Classification of Periodontal and Peri-Implant Diseases and Conditions [29], possessing at least 16 natural teeth, and capable of understanding and signing informed consent and follow study instructions.

#### Exclusion criteria

Individuals treated with antibiotics in the preceding 4 weeks or currently undergoing antibiotic therapy, regular consumers of xylitol, coconut, or coconut derivatives or CHX, patients who had received dental prophylaxis within the last 6 months, pregnant and breastfeeding individuals, patients with allergies to coconut, coconut-derived products and CHX, those with uncontrolled systemic diseases or current use of medications such as phenytoin, cyclosporine, immunosuppressants or anticoagulants, and those with active systemic diseases (e.g., cancer or infectious diseases other than periodontitis) or history of chemotherapy or radiotherapy to the head and neck area.

### Intervention protocols

- Baseline (T1): Diagnostic procedures for determining the presence of periodontal disease based on the 2017 World Workshop on the Classification of Periodontal and Peri-Implant Diseases and Conditions [27] were performed by a blinded specialist in periodontics (SPL). Saliva and crevicular fluid sample collection was collected by the same periodontist. Each patient received oral health instructions, including the Modified Bass brushing technique [30, 31] to be performed three times daily and flossing after night brushing, to ensure consistency across participants and groups. They were also instructed to begin rinsing with their assigned mouthwash.
- One month after baseline (T2): Saliva and crevicular fluid samples collected prior to treatment. At this point, non-surgical periodontal therapy was performed by three trained dental hygienists following the European Federation of Periodontology (EFP) clinical guidelines [32]. Step 1 involved professional mechanical plaque removal and control of plaque-retentive factors, while Step 2 included subgingival periodontal instrumentation using hand and powered ultrasonic instruments [32].
- One Month after periodontal therapy (T3): saliva and crevicular fluid samples were collected. Each patient was instructed not to eat, wash, smoke or rinse their teeth at least one hour before saliva sampling at each visit.

### Standardization / training

Prior to the study, the dental hygienists involved in the study were standardized and trained by SPL, emphasizing standardization of techniques, precision in instrumentation, and strict adherence to study protocols to ensure consistency and reliability across all procedures. Patients were provided with oral health instructions and instructed to discontinue the use of the mouthwash.

### Primary study outcomes assessed

#### Oral Microbiome from saliva and GCF

One 8 mL tube of non-stimulated saliva was collected for each patient at T1, T2 and T3, and were immediately stored at -80ºC for later analysis.

GCF was obtained at T1, T2 and T3 using sterile #30 absorbent paper points (Henry Schein, NY, USA) that were inserted in the gingival sulcus of 3 different teeth that presented between 4 and 6 mm PPD for 30 s and then submerged in 1 mL of RNA later (Qiagen, Venlo, Netherlands). Eight paper points were used for each patient. Samples were stored at -80ºC for later analysis.

#### Bacterial DNA extraction

Saliva samples were thawed at room temperature, transferred to 50 mL tubes, and centrifuged at 13,000 rpm for 10 min at 4 °C. The supernatant was discarded, and the pellet was resuspended in 100 µL of nuclease-free water (Thermo Fisher Scientific, USA) and transferred to a 2 mL Eppendorf tube. For bacterial lysis, 5 µL of 20 mg/mL lysozyme, 1.25 KU/mL lysostaphin, and 3 KU/mL mutanolysin (Sigma-Aldrich, USA) was added and incubated at 37 °C with shaking for 1 h. DNA extraction was performed using the MasterPure™ Complete DNA & RNA Purification Kit (Epicentre, USA), and the extracted DNA was resuspended in 35 µL of TE buffer (10 mM Tris-Cl, 1 mM EDTA, pH 8.0) and stored at -20 °C.

For GCF samples, vortexing for 4 min detached bacteria from paper points that were squeezed against the tube walls and removed. The tubes were centrifuged at 13,000 rpm for 30 min at 4 °C. The precipitates were treated with 30 µL of enzyme cocktail and incubated at 37 °C for 1 h. After adding 2 µL of proteinase K, the samples were placed on ice for 10 min, followed by centrifugation for 5 min at 4000 g at 4 °C. DNA extraction was carried out using the AllPrep DNA/RNA Kit (Qiagen), and the DNA was eluted in 30 µL of EB buffer (10 mM Tris-Cl, pH 8.5) and stored at -20 °C. An extraction control was included for each series.

#### Library Preparation

The DNA concentration of each sample was determined using the Qubit dsDNA HS Assay Kit (Invitrogen, USA) to prepare a 5 ng/µL dilution for library preparation. Two PCR reactions were required for 16 S rRNA metabarcoding.

The first PCR amplified the V3-V4 region of 16 S rRNA using the primers:

Forward: 5’TCGTCGGCAGCGTCAGATGTGTATAAGAGACAGCCTACGGGNGGCWGCAG’3.

Reverse: 5’GTGACTGGAGTTCAGACGTGTGCTCTTCCGATCTGACTACHVGGGTATCTAATCC’3.

For each sample, a mixture of 1.25 µL of each primer (10 µM), 12.5 µL NZYTECH polymerase, 5 µL DNA (5 ng/µL), and 5 µL nuclease-free water were prepared, including a negative control. The amplification program was 95 °C for 5 min, 25 cycles of 95 °C for 30 s, 50 °C for 45 s, and 75 °C for 45 s, with a final step at 72 °C for 5 min. PCR products were checked by electrophoresis (550 bp) and purified using the AMPure XP system (Beckman Coulter, USA). After two ethanol washes, DNA was eluted in 50 µL of EB buffer.

For library preparation, the Nextera XT Index Kit (Illumina, USA) was used. A second PCR added the indexes for sequencing, with a mixture like the previous reaction but replacing primers with 2.5 µL of indexes. The conditions were 95 °C for 3 min, 5 cycles of 95 °C for 30 s, 60 °C for 45 s, and 72 °C for 45 s, followed by 5 min at 72 °C. The products were verified via electrophoresis on a 1% agarose gel and purified again using AMPure XP. Libraries were quantified using the dsDNA HS Assay Kit (Invitrogen, USA).

#### Sequencing

For each sample, a specific volume was taken based on the sample with the lowest concentration to obtain an equimolar pool in which all samples were equally represented. The final pool was successively diluted to reach a final concentration of 12 pM. Finally, 80% of the pool was mixed with 20% of Phix 12 pM (Illumina, USA). The samples were sequenced using the Illumina MiSeq v3 2 × 300 paired-end kit (Illumina, USA) and the MiSeq platform (Illumina USA).

### Interleukin-6 and TNF-α from saliva

One 8 mL tube of non-stimulated saliva was collected for each patient at T1, T2 and T3, and were immediately stored at -80ºC for later analysis.

To determine the levels of IL-6 the samples were centrifuged at 13,000 g for 5 min at room temperature. The supernatant was measured following the manufacturer’s instructions, using the Human IL-6 DY206 ELISA kit from DuoSet (Minneapolis, USA).

To determine the levels of TNF-α, the samples from the patients were concentrated using the Savant SpeedVac SPD121P from Thermo Fisher to facilitate the detection of the protein. Quantification was carried out with the Human TNF-alpha DY210 ELISA kit from DuoSet (Minneapolis, USA). All measurements were performed on the Tecan Infinite^®^ 200 PRO NanoQuant at 450 nm with correction at 570 nm in duplicate. In both cases the protein content was expressed in pg/ml.

### Statistical analysis

After sequencing the 16 S rRNA gene, FASTQ files were generated, and their quality was verified using FastQC. Quality filtering and analysis were performed using QIIME2, where DADA2 was applied to remove primers, adapters, chimeras, and taxonomic groups found in controls. Amplicon sequence variants (ASVs) and taxonomic assignments were generated, and rarefaction curves were produced to assess sample diversity coverage.

Taxonomic assignments were made using the SILVA 138.1 reference database, grouping ASVs and calculating relative abundances for each sample at various taxonomic levels. Bar charts were created using Phyloseq and ggplot2 in R, with unclassified ASVs labeled by their last known taxon and “NA.” ASVs with an abundance of less than 0.01% or present in fewer than 30–50% of samples were filtered out for relative abundance diagrams.

Alpha diversity was assessed using QIIME2 with ACE (Abundance-based Coverage Estimator), Fisher, Shannon, and Simpson indices. The Wilcoxon rank-sum test was used to compare data across different time points and treatments. Beta diversity was also analyzed using QIIME2 with Bray-Curtis, Jaccard, Jensen-Shannon, and Weighted UniFrac indices to study sample composition similarities.

A normalized CLR (Centered Log-Ratio) abundance analysis was performed at family, genus, and species levels using ggplot2 in R, with values transformed to a logarithmic scale for group comparisons. Box plots were created, and the Wilcoxon rank-sum test was applied. Lastly, the subgingival microbial dysbiosis index (SMDI) was calculated at the genus level based on the Chen et al. study [33]. 

A Brunner-Langer model for longitudinal data was employed to evaluate and compare changes in Interleukin levels across follow-up periods between groups, using the ATS statistics to determine main effects and interactions. For specific time-point comparisons, the Mann-Whitney test with Bonferroni correction was applied, while the Wilcoxon test with Bonferroni correction was used for within-group comparisons over time. Baseline group homogeneity was assessed using the Kruskal-Wallis test. A significance level of 5% (α = 0.05) was applied to all analyses.

### Preparation of the rinses

For the CO rinses, four commercial coconut oils were analyzed using gas chromatography to assess their composition. The coconut oil with the highest lauric acid content was selected, resulting in the use of pure virgin coconut oil with 47.92% C12:0 for the study. (Superalimentos MundoArcoiris, Girona, Spain)

For the CHX rinses, a commercial 0.12% CHX solution (Lacer, 08290, Barcelona, Spain) was chosen, with three drops of concentrated coconut flavoring (Nature’s Flavour, Alphapower Food, Gauting, Germany) added to provide coconut flavor.

For the placebo rinses, water was used as the base, with the same coconut flavoring added as in the CHX group to maintain consistency.

### Sample size calculation

The sample size was calculated based on similar studies evaluating the effects of essential-oil mouthrinse on subgingival periodontopathogens [34]. The calculations carried out indicate that a minimum of 5 patients per group for a t test to reach 80% power at 95% confidence level were necessary to ensure statistical reliability and avoid overlooking significant results. Since this study includes a third group, adjustments for multiple comparisons were necessary using the Bonferroni criterion. Additionally, anticipating a dropout rate of 20%, the sample size was increased to 10 patients per group.

### Randomization and group allocation

Participants were randomly assigned to one of three groups in a 1:1:1 ratio using block randomization (block size of 3) performed by (AD) to ensure balanced sample sizes. Opaque 250 ml marked containers for measuring rinse volume, standardized toothbrushes, and toothpaste were distributed to participants by (IFM), who was unaware of the contents and distinct from the person collecting samples.

Each participant received standardized toothbrushes (Gum Classic, SUNSTAR Suisse, Switzerland) toothpaste and dental floss (Gum, SUNSTAR Suisse, Switzerland) and dental floss, and was instructed not to use any additional dental products. Participants were directed to rinse vigorously with 5 ml of the allocated mouthwash after brushing at night as follows:

- **Group 1 (CO Group)**: Coconut oil for 10 min, as described in similar studies [25, 34–37].
- **Group 2 (CHX Group)**: 0.12% CHX solution with coconut flavor for 1 min.
- **Group 3 (Placebo Group)**: Coconut-flavored water for 1 min.

Participants, clinicians, study personnel, and the statistician were all blinded to group assignments.

## Results

A total of 30 patients were enrolled and completed all study visits. The cohort included 15 females and 15 males, with an age range of 33 to 72 years (median age: 53.5 years). (Table 1) This balanced sex distribution and relatively narrow age spread facilitate comparison across groups while minimizing demographic biases.

**Table 1:** Demographics. Coconut oil (CO), chlorhexidine (CHX), SD (Standard deviation)

	CO (*N* = 10)	CHX (*N* = 10)	Placebo (*N* = 10)	Total (*N* = 30)
Age (years)				
Mean	53.3	51.2	53.8	52.8
SD	9.57	13.09	9.82	10.35
Median	54	50	57	53.5
Minimum	41	33	37	33
Maximum	67	72	65	72
Gender (N [%])				
Male	5 (50)	5 (50)	5 (50)	15 (50)
Female	5 (50)	5 (50)	5 (50)	15 (50)
Race (N [%])				
White	10 (100)	10 (100)	10 (100)	30 (100)

### Bacterial alpha diversity of the samples

**Fig. 1:**
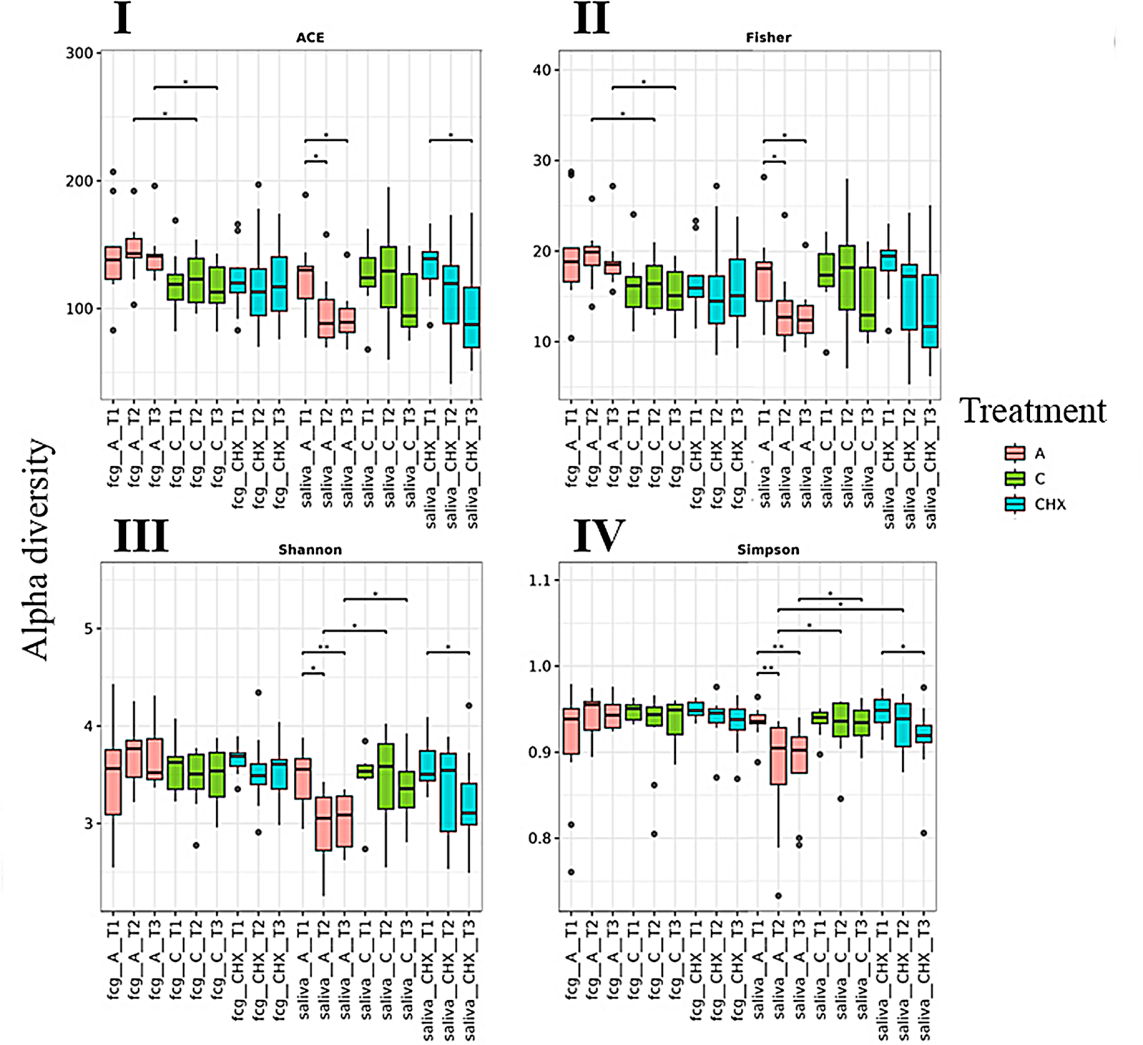
Alpha-diversity of gingival crevicular fluid (FCG) and saliva samples grouped according to treatment type and sample collection time using ACE (Abundance-based Coverage Estimator) (I), Fisher (II), Shannon (III), and Simpson (IV) indices. Each color corresponds to a treatment type: **A**) Red color: treatment with placebo, **C**) green color: treatment with coconut oil; and CHX) blue color, treatment with Chlorhexidine. T1, T2 and T3 represent the time of sampling. Wilcoxon rank-sum statistical test was used: * *p* < 0.05, ** *p* < 0.01

In the GCF samples, the CO group showed relatively stable diversity across time in all indices. The Shannon and Simpson indices indicated that diversity remained stable over time in all treatments. (Fig. 1).

In the saliva samples, all treatments showed similar alpha diversity values (Fig. 1). In the CO group, a notable reduction, although not statistically significant, in diversity was observed over time in the ACE, Fisher, and Shannon indices. However, in the CHX treatment, a statistically significant decrease in diversity was seen in the ACE, Shannon, and Simpson indices over time.

### Bacterial Beta diversity of the samples

**Fig. 2:**
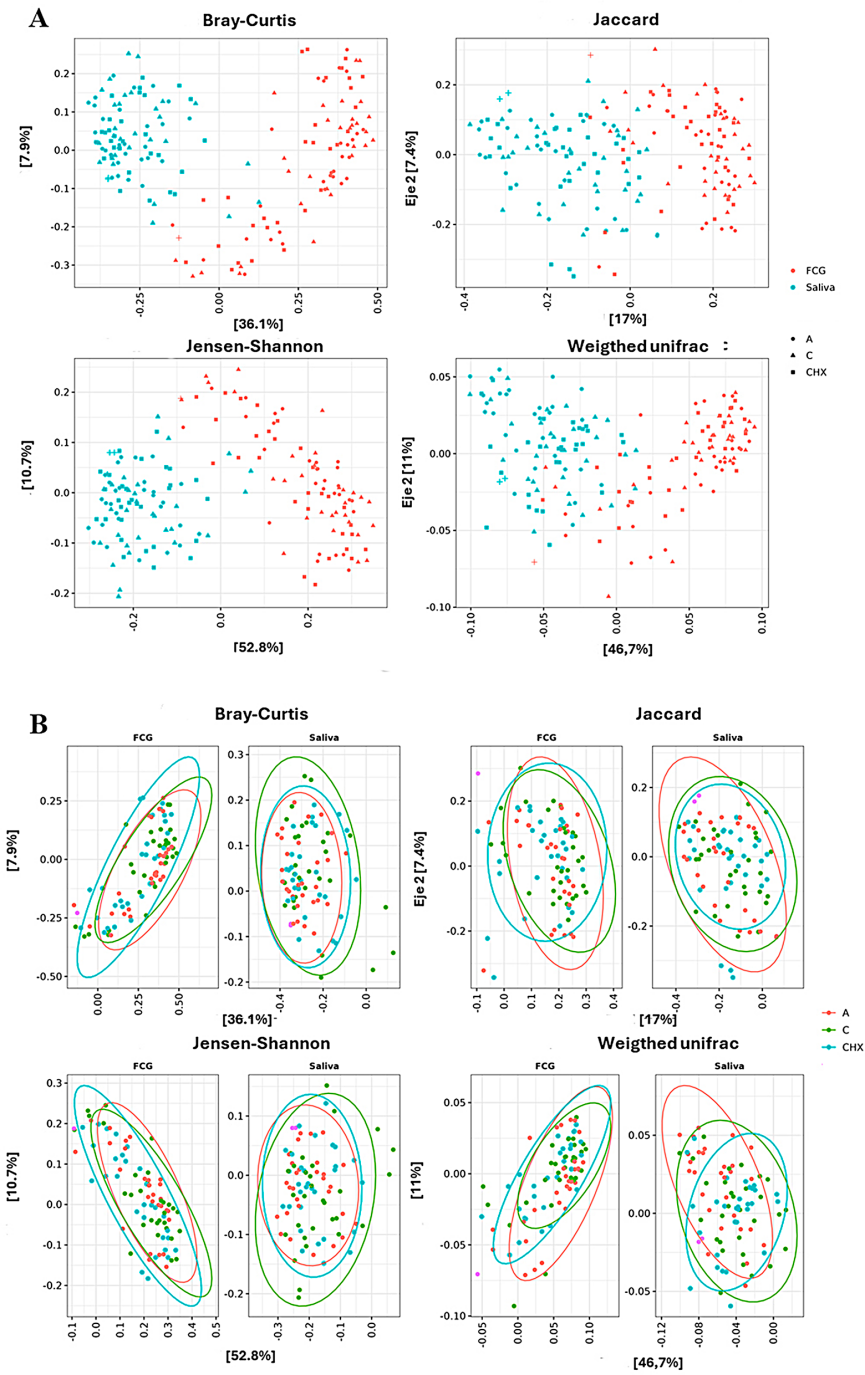
Beta-diversity of samples. **A**) Beta-diversity of each of the samples analyzed. Each color belongs to a type of sample. Gingival crevicular fluid (GCF): Red color. Saliva: blue color. The treatments were differentiated using symbols. Treatment A: placebo, Treatment C: Coconut oil, Treatment CHX: chlorhexidine. **B**) Beta-diversity of the samples separated according to the type of sample (GCF and saliva). Each color belongs to a type of treatment. **A**) Red color: treatment with placebo, **C**) green color: treatment with coconut oil; and CHX) blue color, treatment with Chlorhexidine. In both cases, the Bray-Curtis, Jaccard, Jensen-Shannon and Weighted unifrac indices were used

A clear separation between GCF and saliva samples was observed, indicating that the microbial composition of each of them is different (Fig. 2A). When separately studying saliva and GCF samples (Fig. 2B), similarity in bacterial diversity between treatments was observed.

### Analysis of the oral Microbiome

**Fig. 3:**
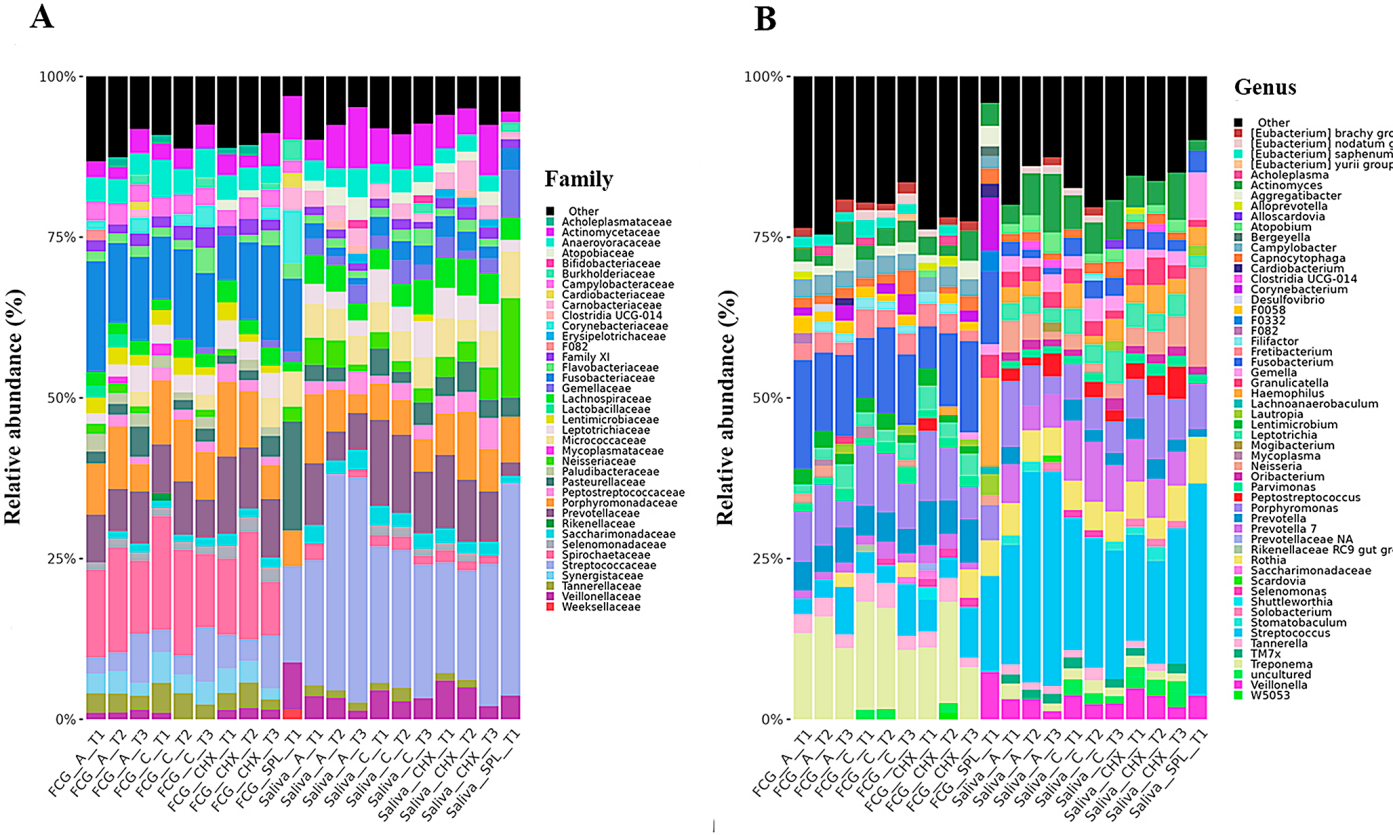
Bacterioma of the pooled samples obtained from gingival crevicular fluid (GCF) and saliva samples from patients with periodontitis determined by 16 S rRNA metabarcoding. They were grouped according to the type of sample, the type of treatment and the time of sampling **A**) at the family level and **B**) at the genus level. The samples analyzed were based on a treatment with placebo (A), Coconut oil (C) and (CHX) chlorhexidine, obtaining the samples at different times: T1, T2 and T3

In GCF samples the relative abundance of bacterial families Spirochaetaceae and Fusobacteriaceae predominated, and to a lesser extent, Porphyromonadaceae and Prevotellaceae, while in saliva samples, Streptococcaceae predominated, and to a lesser extent, Porphyromonadaceae and Prevotellaceae (Fig. 3A).

The relative abundance of the bacteriome at the genus level showed an abundance of *Fusobacterium*, *Porphyromona*s, and *Treponema* in GCF samples, while *Streptococcus* predominated in saliva samples, and to a lesser extent, *Porphyromonas* and *Prevotella*. (Fig. 3B)

**Fig. 4:**
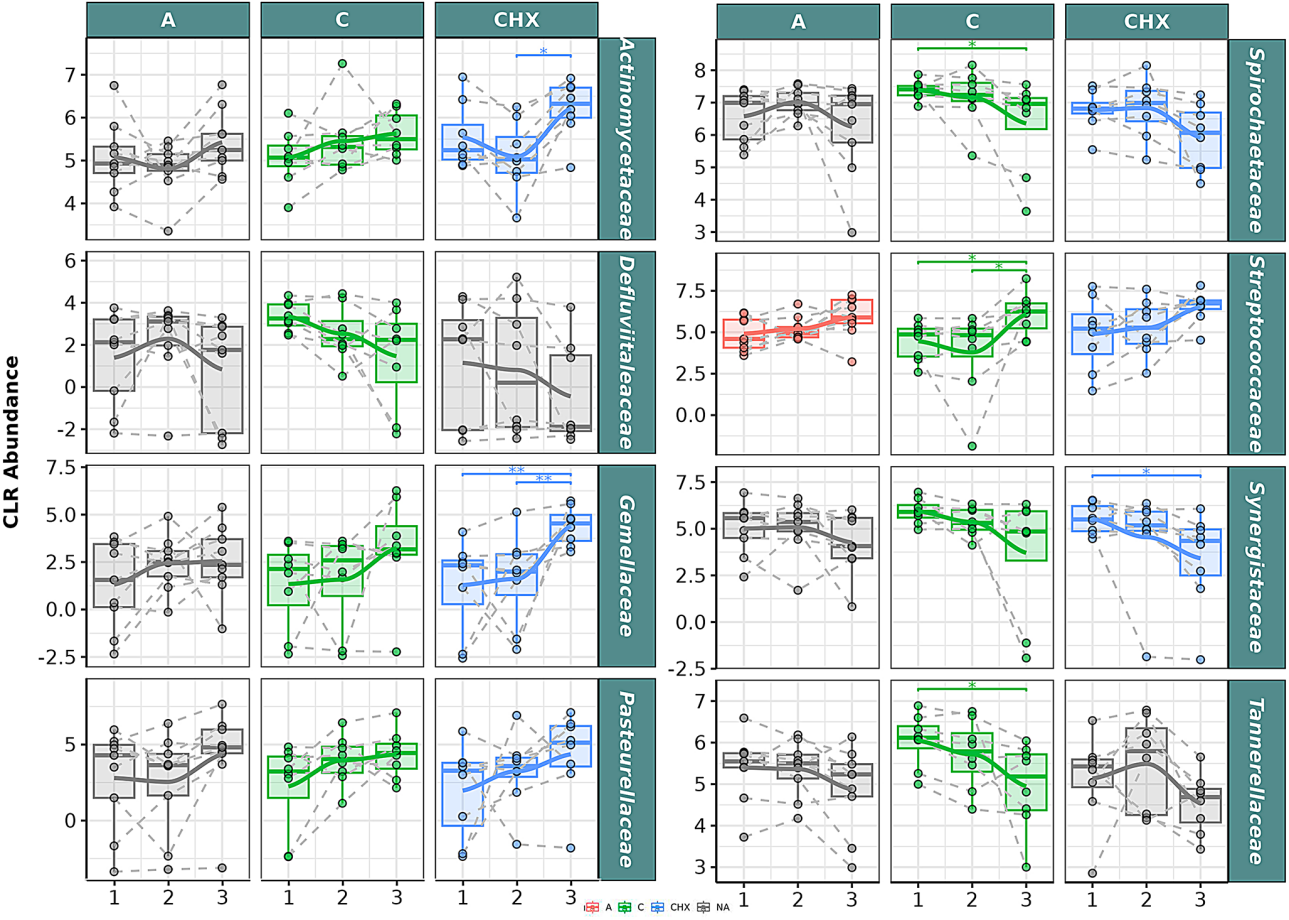
Selected bacterial families present in the oral microbiome of gingival crevicular fluid (GCF) samples from patients with periodontitis. The box plot represents the normalized Centered Log-Ratio (CLR) abundance of each family according to the type of treatment **A**: placebo; **C**: Coconut oil; CHX; and time of sampling (T1, T2 and T3). Each color belongs to a treatment in which there was a significant correlation over time, represented by a line that passes through the boxes: A) Red color: treatment with placebo, **C**) green color: treatment with coconut oil; and CHX) blue color, treatment with Chlorhexidine. Samples from the same patient were connected by dotted lines. The gray color is assigned when no significant correlation was obtained in the treatment. The Wilcoxon rank-sum statistical test was used: * *p* < 0.05, ** *p* < 0.01

In the GCF samples, at the family level, bacteria belonging to Defluviitaleaceae, Spirochaetaceae, Synergistaceae, and Tannerellaceae families had a significant descending correlation in the samples of patients treated with CO over time (Fig. 4). Significant differences were obtained between T1 and T3 for the Spirochaeraceae and Tannerellaceae genera. Regarding Actinomycetaceae, Gemellaceae, Pasteurellaceae, and Streptococcaceae families, a significant positive correlation was found in CO samples, with significant differences between T1 and T3 and between T2 and T3 in Streptococcaceae.

**Fig. 5:**
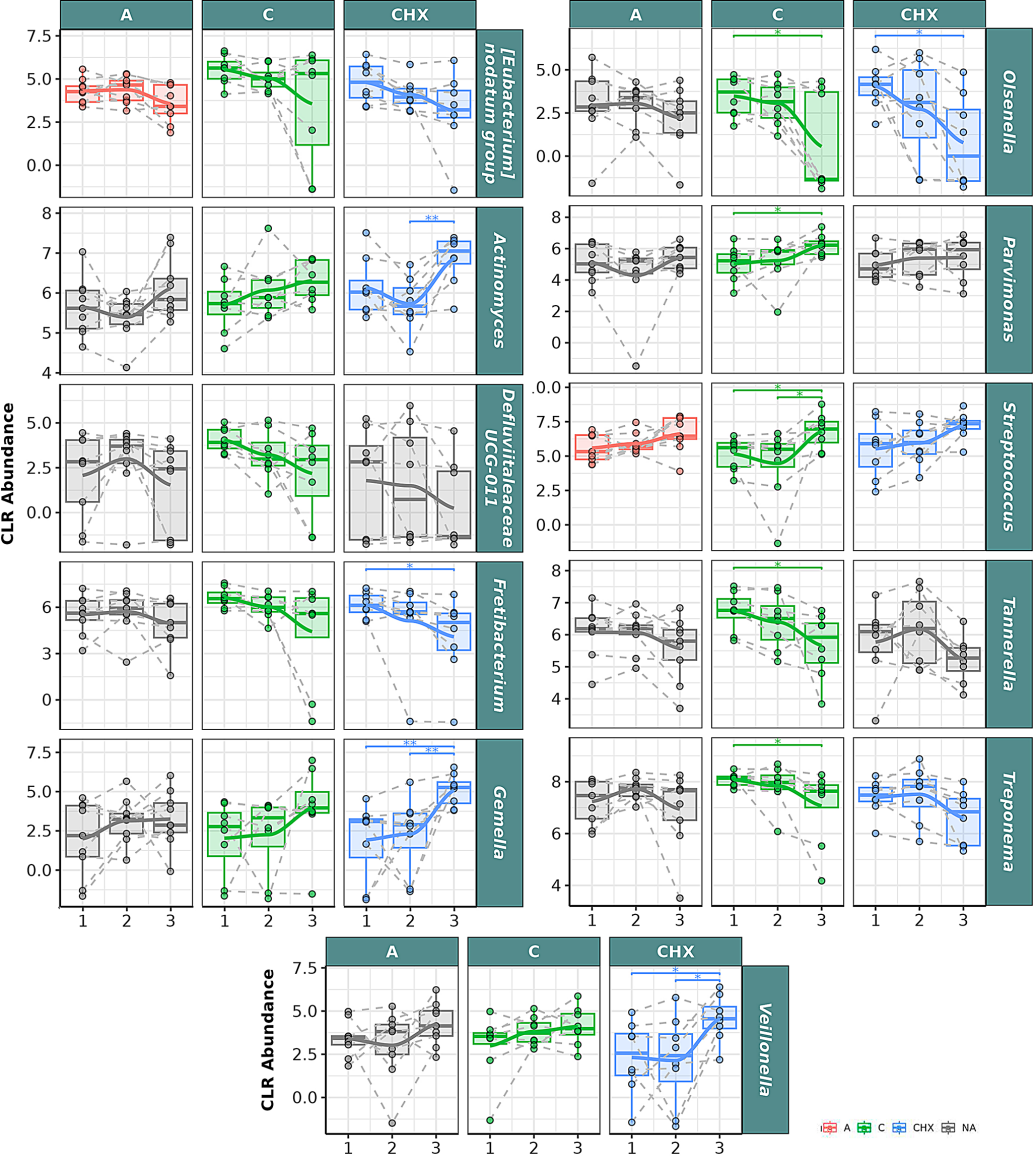
Selected bacterial genera present in the oral microbiome of gingival crevicular fluid (GCF) samples from patients with periodontitis. The box plot represents the Centered Log-Ratio (CLR) abundance of each genus according to the type of treatment (A: placebo; C: coconut oil; and CHX: Chlorhexidine) and time of sampling (T1, T2 and T3). Each color belongs to a treatment in which there was a significant correlation over time, represented by a line that passes through the boxes: A) Red color: treatment with placebo, C) green color: treatment with coconut oil; and CHX) blue color, treatment with Chlorhexidine. The gray color is assigned when no significant correlation was obtained in the treatment. Samples from the same patient were connected by dotted lines. The Wilcoxon rank-sum statistical test was used: * *p* < 0.05, ** *p* < 0.01

In the GCF samples at the genus level (Fig. 5), the abundance of Defluviitaleaceae UCG-011, *Fretibacterium*,* Olsenella*,* Tannerella*,* Treponema*, and the *Eubacterium* group had a significant descending correlation over time in CO. Additionally, Tannerella and Treponema showed significant abundance reduction between T1 and T3. Significant ascending correlation was observed for *Actinomyces*, *Gemella*, *Parvimonas*, *Streptococcus*, and *Veillonella* genera over time, with significant differences between T1 and T3 for *Parvimonas* and between T2 and T3 for *Streptococcus*.

**Fig. 6:**
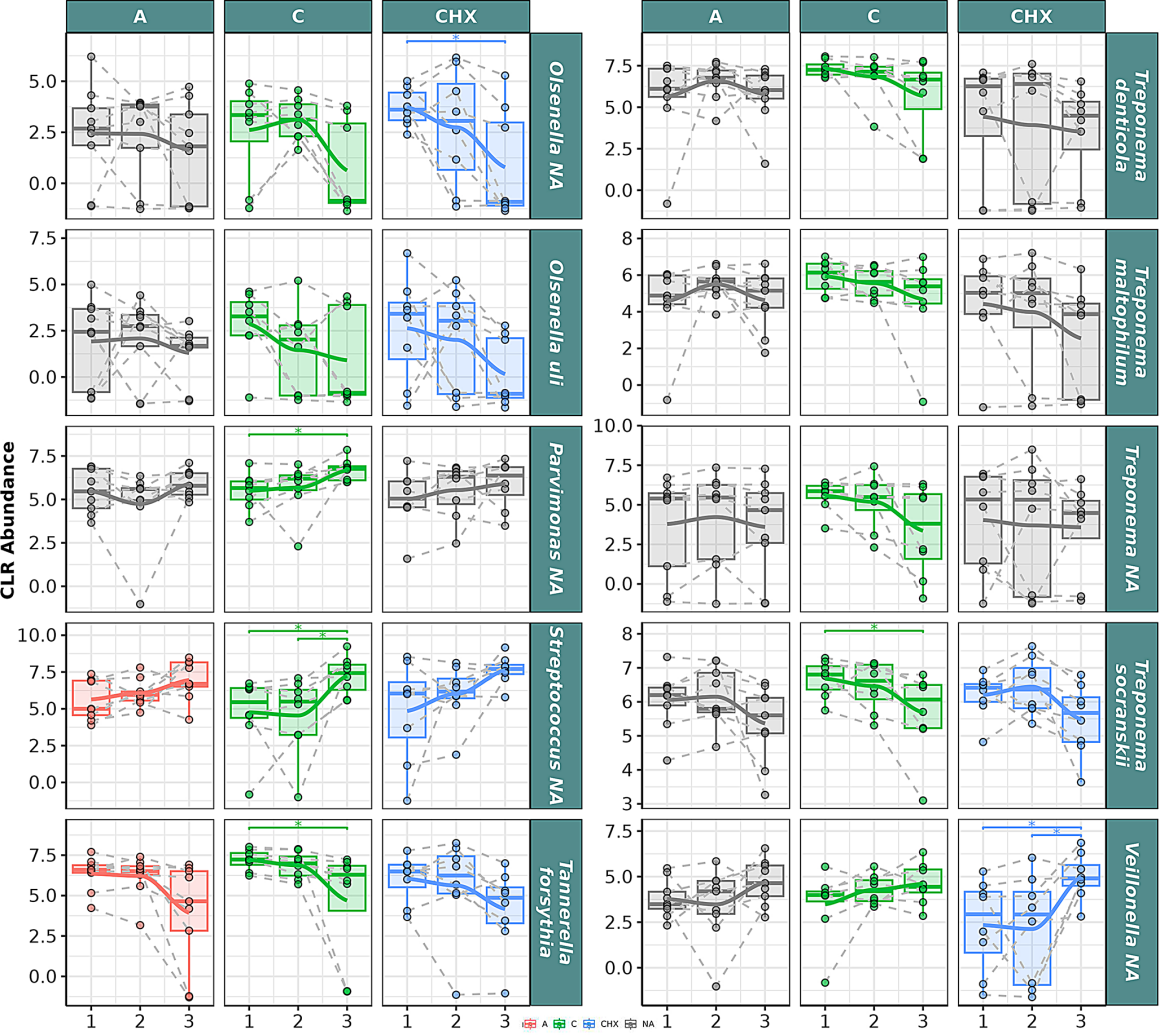
Selected bacterial species present in the oral microbiome of gingival crevicular fluid (GCF) samples from patients with periodontitis. The box plot represents the Centered Log-Ratio (CLR) abundance of each species according to the type of treatment (**A**: placebo, **C**: coconut oil and CHX: chlorhexidine) and time of sampling (T1, T2 and T3). Each color belongs to a treatment in which there was a significant correlation over time, represented by a line that passes through the boxes: **A**) Red color: treatment with placebo, **C**) green color: treatment with coconut oil; and CHX) blue color, treatment with Chlorhexidine. The gray color is assigned when no significant correlation was obtained in the treatment. Samples from the same patient were connected by dotted lines. The Wilcoxon rank-sum statistical test was used: * *p* < 0.05, ** *p* < 0.01

In the GCF samples at the species level (Fig. 6), the species *Fretibacterium* NA, *Olsenella uli*,* Olsenella* NA, *Tannerella forsythia*,* Treponema denticola*,* Treponema maltophilum*,* Treponema* NA, and *Treponema socranskii* showed a significant descending correlation over time in CO, with significant differences between T1 and T3 for *Tannerella forsythia*. Significant ascending correlation was found over time for *Parvimonas* NA and *Streptococcus* NA, with significant increases between T1 and T3 and between T2 and T3 for the latter. Only significant positive correlation over time was observed for *Veillonella* NA.

**Fig. 7:**
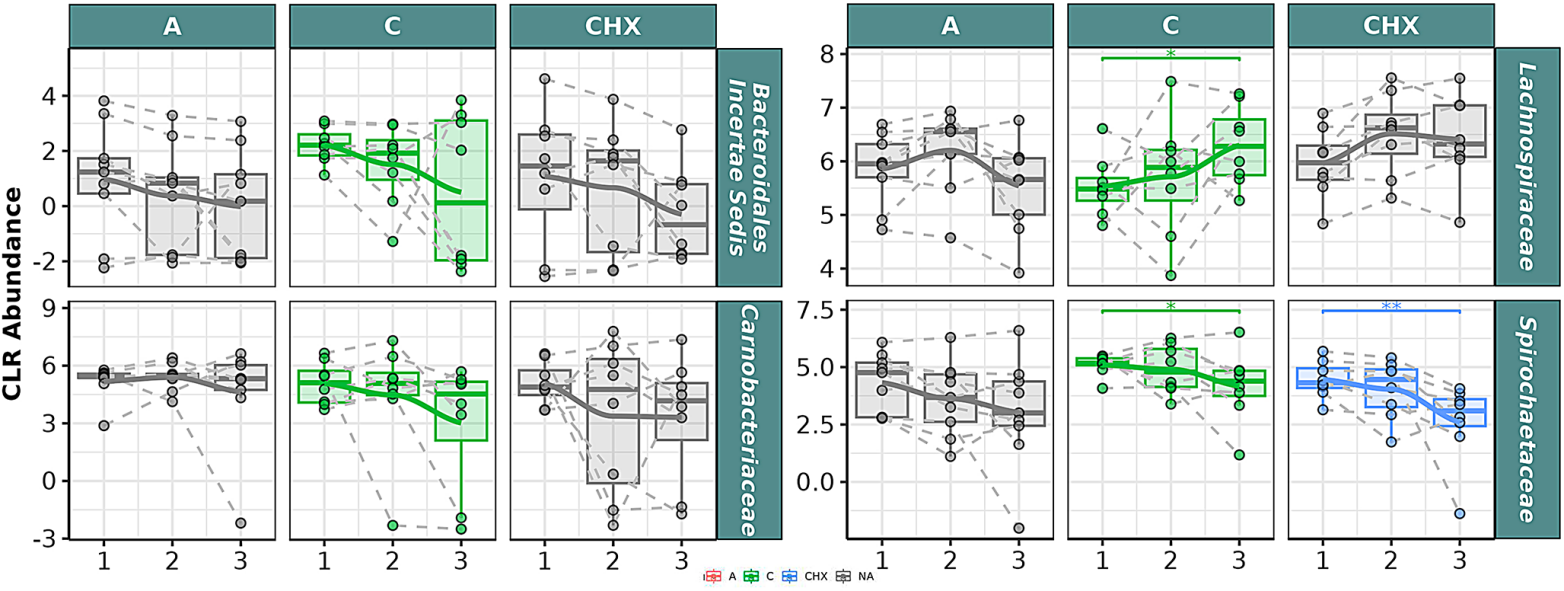
Selected bacterial families are present in the oral microbiome of saliva samples from patients with periodontitis. The box plot represents the Centered Log-Ratio (CLR) abundance of each family according to the type of treatment (**A**: placebo, **C**: coconut oil and CHX: chlorhexidine) and time of sampling (T1, T2 and T3). Each color belongs to a treatment in which there was a significant correlation over time, represented by a line that passes through the boxes: **A**) Red color: treatment with placebo, **C**) green color: treatment with coconut oil; and CHX: blue color, treatment with Chlorhexidine. The gray color is assigned when no significant correlation was obtained in the treatment. Samples from the same patient were connected by dotted lines. The Wilcoxon rank-sum statistical test was used: * *p* < 0.05, ** *p* < 0.01

In saliva samples (Fig. 7), CO had a decreasing effect on Bacteroidales incertae sedis, Carnobacteriaceae, and Spirochaetaceae families, with a significant descending correlation over time, with significant differences between T1 and T3 for Spirochaetaceae. An increase in abundance was observed for Lachnospiraceae over time, with significant ascending correlation and differences between T1 and T3.

**Fig. 8:**
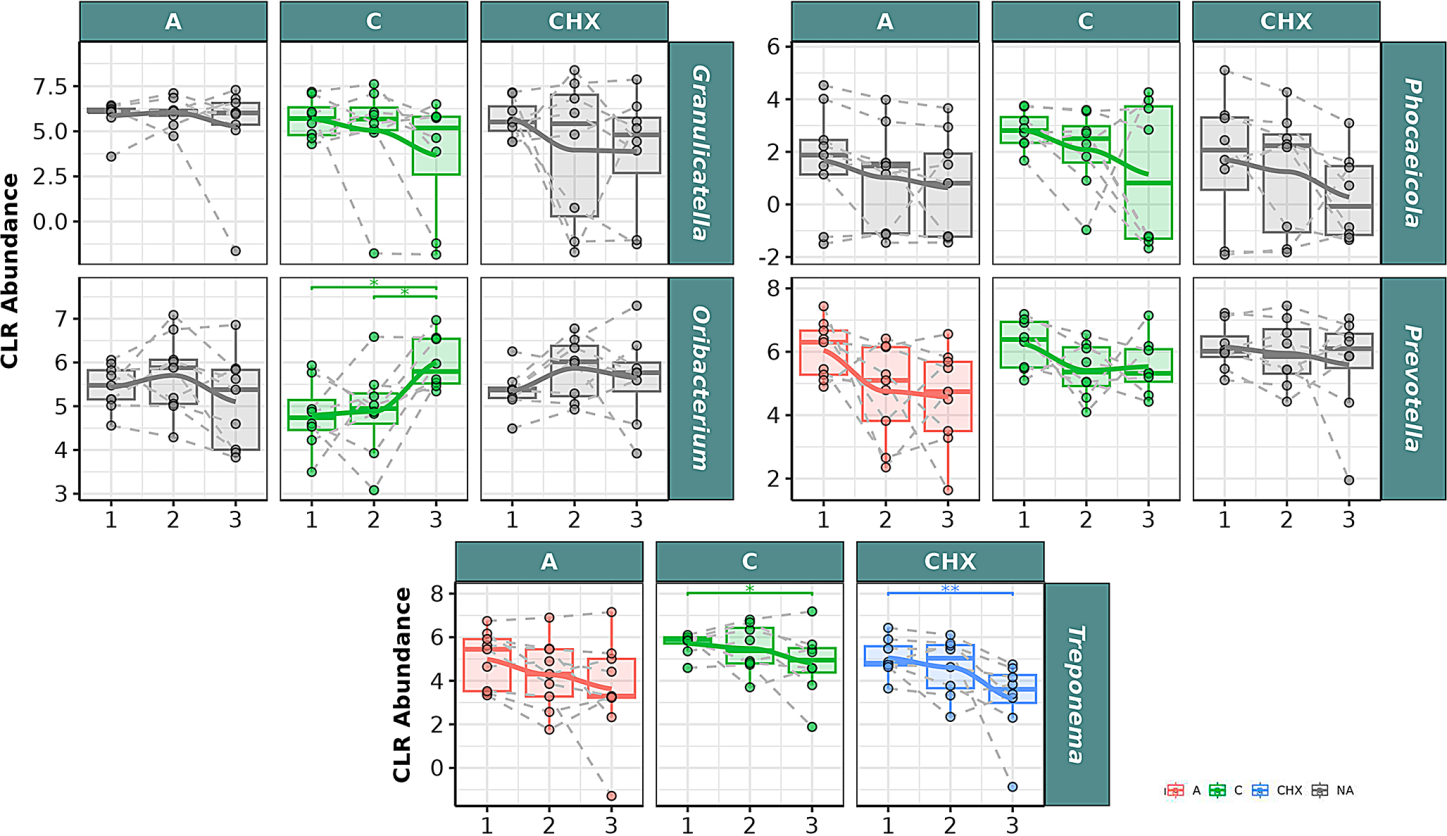
Selected bacterial genera present in the oral microbiome of saliva samples from patients with periodontitis. The box plot represents the Centered Log-Ratio (CLR) abundance of each genus according to the type of treatment (A: placebo, C: coconut oil and CHX: chlorhexidine) and time of sampling (T1, T2 and T3). Each color belongs to a treatment in which there was a significant correlation over time, represented by a line that passes through the boxes: **A**) Red color: treatment with placebo, **C**) green color: treatment with coconut oil; and CHX) blue color, treatment with Chlorhexidine. The gray color is assigned when no significant correlation was obtained in the treatment. Samples from the same patient were connected by dotted lines. The Wilcoxon rank-sum statistical test was used: * *p* < 0.05

In the analysis of the effect of CO on the oral microbiome at the genus level in saliva samples (Fig. 8), significant descending correlation over time was observed for *Granulicatella*,* Phocaeicola*,* Prevotella*, and *Treponema*, with significant differences between T1 and T3. A significant correlation over time was observed for *Oribacterium*, with significant differences between T1 and T3 and between T2 and T3.

**Fig. 9:**
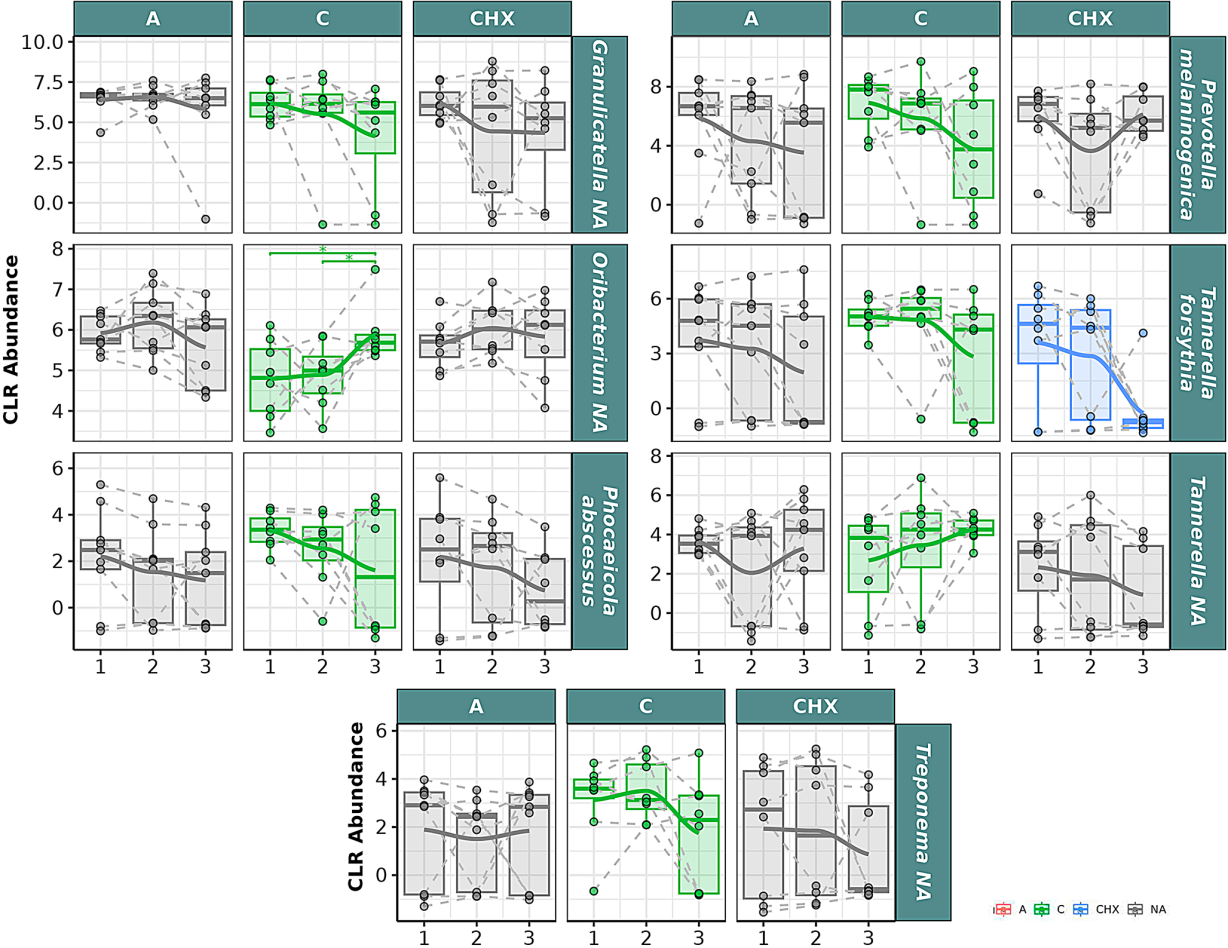
Selected bacterial species present in the oral microbiome of saliva samples from patients with periodontitis. The box plot represents the Centered Log-Ratio (CLR) abundance of each species according to the type of treatment (A: placebo, C: coconut oil and CHX: chlorhexidine) and time of sampling (T1, T2 and T3). Each color belongs to a treatment in which there was a significant correlation over time, represented by a line passing through the boxes: **A**) Red color: treatment with placebo, **C**) green color: treatment with coconut oil; and CHX) blue color, treatment with Chlorhexidine. The gray color is assigned when no significant correlation was obtained in the treatment. Samples from the same patient were connected by dotted lines. The Wilcoxon rank-sum statistical test was used: * *p* < 0.05

In the analysis of bacterial species in saliva samples (Fig. 9), significant descending correlation was observed in the CO group for *Granulicatella* NA, *Phocaeicola abscessus*, *Prevotella melaninogenica*, *Tannerella forsythia*, and *Treponema* NA. Significant ascending correlation over time was observed for *Oribacterium* NA and *Tannerella* NA, with significant differences between T1 and T3 and between T2 and T3 for *Oribacterium* NA.

**Fig. 10:**
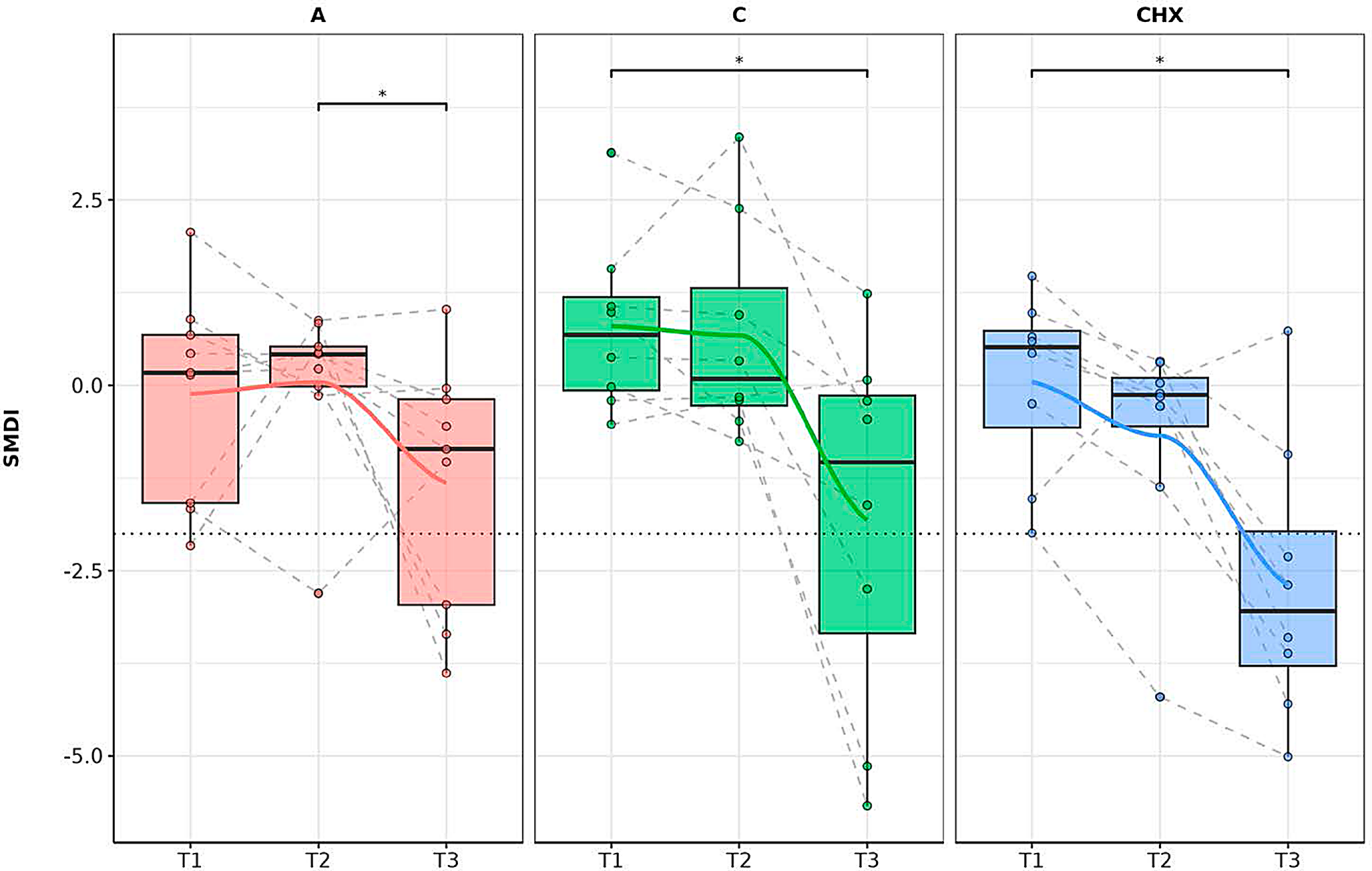
Subgingival dysbiosis index (SMDI) at gender level. The box plot represents the SDMI based on a group of genera associated with subgingival dysbiosis according to the type of treatment (**A**: placebo, **C**: coconut oil and CHX: chlorhexidine) and time of sample (T1, T2 and T3). Each color belongs to a treatment in which there was a significant correlation over time, represented by a line that passes through the boxes: **A**) Red color: treatment with placebo, **C**) green color: treatment with coconut oil; and CHX) blue color, treatment with Chlorhexidine. Samples from the same patient were connected by dotted lines. The Wilcoxon rank-sum statistical test was used: * *p* < 0.05

A significant decrease in SMDI between T1 and T3 was observed for CO and CXH, with a pronounced decrease between T2 and T3, demonstrating a significant shift toward a more balanced microbial profile, while in the placebo group significant differences were only observed between T2 and T3. (Fig. 10)

### Interleukin-6 and TNF-α

The CO group showed a significant decrease in IL-6 levels over the entire period (*p* = 0.021) and between T2 and T3 when comparing CO with placebo (*p* = 0.027).

**Fig. 11:**
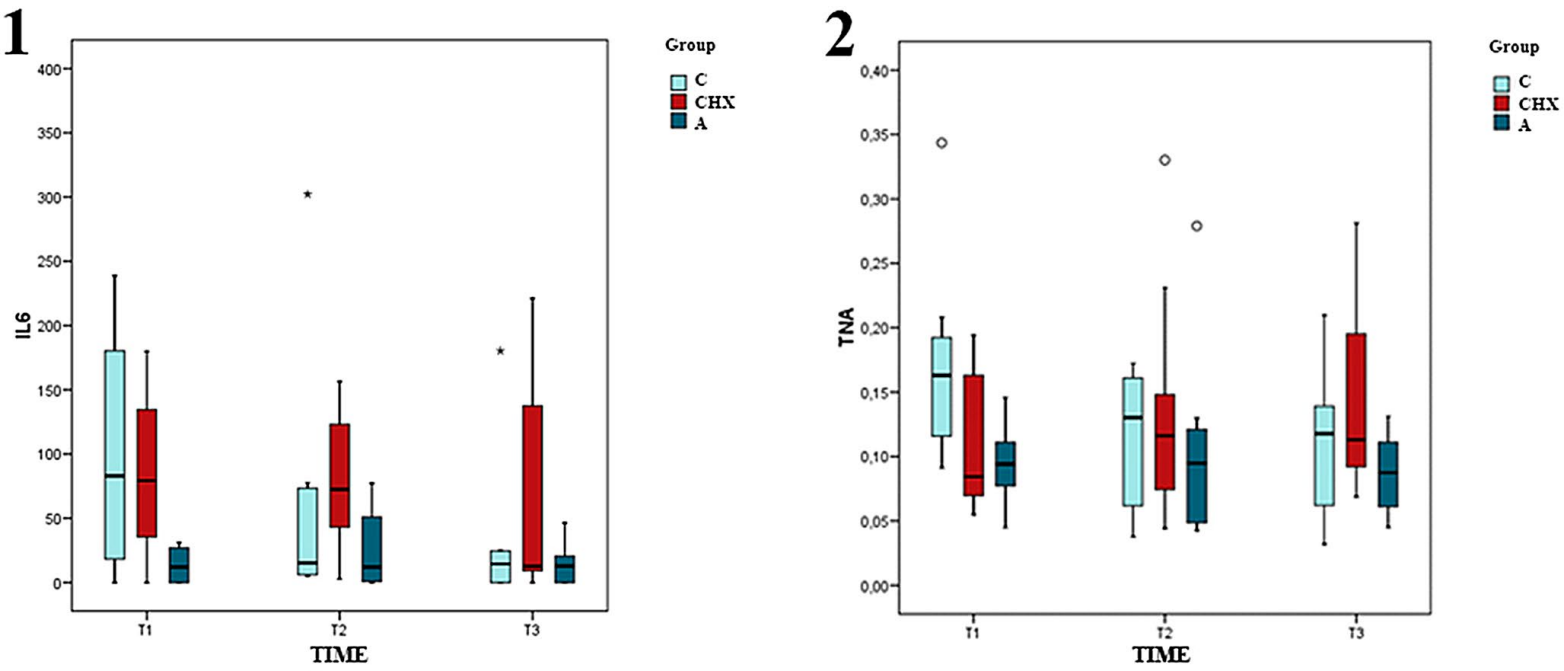
The graph displays the levels of IL-6 (1) and TNF-α (2) according to the type of treatment (A: placebo, C: coconut oil and CHX: chlorhexidine) and time of sampling (T1, T2 and T3)

Similarly, a significant reduction in TNF-α was observed only in the CO group between T1 and T3 (*p* = 0.021) and between T1 and T3 when comparing CO and CHX, favoring CO (*p* = 0.045). (Table 2) (Fig. 11).

**Table 2:** Coconut oil (CO), chlorhexidine (CHX), SD (Standard deviation)

Outcomes			CO	CHX	Placebo	CO vs. CHX	CO vs. Placebo	CHX vs. Placebo
IL6	T1 vs. T2	Mean	-47,6	-5,93	12,5	-41,7	-60	-18,4
		SD	77,2	80,3	32,8	35,2	26,5	27,4
		P value	0,222	1	1	1	0,189	0,945
	T1 vs. T3	Mean	-72,4	8,72	0,61	-81,1	-73	8,11
		SD	67,7	161,3	24,5	55,3	22,8	51,6
		P value	**0**,021*	1	1	0,741	1	1
	T2 vs. T3	Mean	-24,8	14,7	-11,8	-39,5	-13	26,5
		SD	42,2	141,9	38,4	46,8	18	46,5
		P value	0,141	1	1	1	0.027*	1
TNA	T1 vs. T2	Mean	-0,05	0,02	0,01	-0,07	-0,06	0,01
		SD	0,07	0,09	0,08	0,03	0,03	0,04
		P value	0,177	1	1	0,189	0,369	1
	T1 vs. T3	Mean	-0,06	0,03	-0,01	-0,07	-0,05	0,03
		SD	0,06	0,08	0,04	0,03	0,02	0,03
		P value	**0**,021*	1	1	0.045*	1	1
	T2 vs. T3	Mean	0	0,01	-0,02	-0,01	0,01	0,02
		SD	0,05	0,09	0,07	0,03	0,03	0,03
		P value	1	1	1	1	0,156	1

^*p<0,05^

## Discussion

To the best of our knowledge, this study represents the first randomized clinical trial to investigate the effects of coconut oil on the oral microbiome and inflammatory response in patients with periodontitis. CO is composed of fatty acids such as lauric acid and monolaurin, which have antibacterial activity. Although fatty acids antimicrobial effects on various bacteria are well documented [17, 23, 38–42], most systematic reviews and meta-analyses evaluating oil pulling focus on clinical outcomes rather than its direct impact on the oral microbiome [25, 43, 44]. The findings on the present study demonstrate that CO treatment significantly modulates the oral microbiome, promoting a shift toward a healthier microbial profile, while also reducing key inflammatory markers. However, the main limitation of this study is its small sample size, indicating the need for larger-scale research to obtain more conclusive results. In patients suffering from periodontitis, there is a significant dysbiosis of the oral microbiota, marked by an increase in certain bacterial species, particularly in the subgingival sulcus. This includes the most pathogenic bacteria of Socransky’s red complex: *Porphyromonas gingivalis*, *Tannerella forsythia*, and *Treponema denticola* [45].

The most effective treatments for combating the disease are mechanical, both surgical and non-surgical, and these can be complemented with antiseptic compounds to control the bacterial load before, during, and after treatment to enhance its effectiveness [15, 46]. One of the most used options is CHX, a synthetic chemical compound with potent antibacterial effects. However, it has been reported to have side effects such as dental staining and taste alteration, thereby limiting its suitability for prolonged use [15]. Rinsing with essential oils such as CO, also known as oil pulling, has shown to be an alternative to CHX due to its antibacterial and anti-inflammatory properties with minimal side effects [47].

Unlike conventional oral rinses designed for 1–2 min of use, oil pulling requires a longer duration to maximize its emulsifying and saponifying actions, which are essential for reducing plaque adhesion and bacterial coaggregation [48]. The extended rinsing time for CO compared to CHX and placebo used in the present study aligns with findings from other studies demonstrating the enhanced effectiveness of oil pulling when performed over prolonged periods [47, 49, 50]. While CHX can yield comparable reductions in bacterial load with a shorter rinsing time, the results from the present study indicate that CO may confer additional anti-inflammatory benefits, likely related to the direct action of lauric acid on proinflammatory cytokines. Although some studies also report that CHX reduces gingival inflammation, this effect may predominantly stem from decreasing bacterial load rather than directly targeting cytokines [51–53]. This study, based on next-generation sequencing technologies, allowed for a detailed analysis of whether there is a significant decrease in pathogenic species and an increase in primary colonizers following different treatments, particularly in GCF samples.

When studying alpha diversity, a measure of the compositional complexity within a specific site or community [54], it was observed that at the start of the treatment, the bacterial diversity values were similar in both saliva and GCF samples, contrary to what was observed by Kim et al. [55]. For the CO treatment, there was a decrease in saliva samples, but not a significant variation over time. This suggests that there may be a general decrease in bacterial quantity, reducing dental plaque, while the proportion of organisms that make up the oral microbiota remains similar. This was observed in greater detail when studying the bacteriome at different taxonomic levels.

Regarding beta diversity, used to describe taxonomical differences between samples [54], there was a clear difference between the oral microbiome composition in GCF and saliva samples, consistent with the findings of Kim et al. [55]. However, the oral microbiome during the different treatments did not show relevant differences, although it would be interesting to observe the effects of treatments over different time periods.

In the GCF samples, a decrease was observed in two bacterial species belonging to Socransky’s red complex, *Tannerella forsythia* and *Treponema denticola*, a group of bacteria central to driving the dysbiotic process underlying periodontitis [56], which was also reported in another study [57].

*Treponema denticola* is recognized as one of the primary etiological agents of periodontitis, owing to its numerous virulence factors, including high motility and chemotaxis, synergistic interactions with other periodontal pathogens, production of cytotoxic metabolites, robust biofilm formation, and cell wall proteins that disrupt host defenses [58].

In contrast, the role of *Tanerella forsythia* in periodontitis has been more recently elucidated through the identification of six KLIKK proteases, which actively degrade the proteins of the gingival cellular tissue during disease progression [59].

However, no studies were found showing a significant effect of CO on *T. forsythia*, although a significant decrease in this species was observed when using essential oils, especially in periodontal pockets [60], and further investigation into the effects of CO on these two pathogens in cellular models is needed.

Other bacteria showing a decrease in abundance when treated with CO include *Porphyromonas gingivalis*, *Eubacterium nodatum*,* Treponema socranskii*, and *Treponema maltophilum*, all associated with periodontitis as reported in the literature [60, 61].

In our study, a decrease in *Olsenella uli* and *T. maltophilum*, present in periodontal pockets [62], was also observed. However, there is no existing literature demonstrating the effect of CO on these bacteria, making the findings of this study particularly interesting, especially for *T. maltophilum*, since CHX treatment did not yield significant differences for this bacterium.

For the genus *Fretibacterium* and the species *Defluviitaleaceae UCG-011*, a decrease in abundance was observed with CO treatment. Studies have shown an abundance of these bacteria in patients with periodontitis, making their reduction with coconut oil treatment noteworthy [63]. 

The subgingival microbiota in a healthy state is composed of bacteria from the genera *Streptococcus*,* Actinomyces*,* Gemella*, and *Veillonella* [64, 65]. When periodontal disease occurs, members of these genera are displaced by pathogenic species. Treatment with CO showed an increase in these beneficial bacteria. This is significant because it suggests that CO not only directly reduces some pathogenic species but also promotes the growth of bacteria associated with oral health, indicating a return to a more balanced, less pathogenic state. For instance, some Streptococcus species can be linked to periodontitis, while others can exhibit activity capable of displacing bacteria from Socransky’s orange or red complex, such as *T. denticola* or *T. forsythia* [5, 66].

On the other hand, CO had an unexpected effect on the genus *Parvimonas*. *P. micra* is known to inhabit the subgingival cavity and act as a pathogen in periodontitis, being one of the most predominant species [67]. Thus, an increase in these bacteria with CO treatment may not be beneficial.

Regarding the effect of CO on the oral microbiota in saliva samples, a decrease in *Phocaeicola*,* Prevotella*, and *Treponema* was observed, with the specific species being *P. abscessus*,* P. melaninogenica*, and *T. forsythia*. These genera have been identified as some of the most abundant in the saliva of patients with periodontitis [68], although the presence of *T. forsythia. P. melaninogenica* is typically classified as an oral commensal, some studies found a high abundance of this species in subgingival areas of periodontitis patients [69]. While *P. abscessus* has been isolated from brain abscesses [70], it has been linked to periodontal disease [71]. Therefore, the observed decrease in these species with CO use is significant.

For the genera *Granulicatella* and *Oribacterium*, an increase in abundance was observed during CO treatment. *Granulicatella* is typically associated with good oral health in the subgingival region [72]. However, *Oribacterium* is a pathobiont present in both supragingival and subgingival microbiomes of periodontitis patients [73]. Therefore, the increase in these bacteria due to CO treatment could be less beneficial, though these results should be confirmed with a larger study.

Non-surgical periodontal therapy performed may also influence these results, as significant differences were found between the start of the treatment (T1) and one month after dental cleaning (T3). Dental cleanings in healthy patients have been shown to reduce bacterial quantity while maintaining the proportional balance of different bacterial species, which may explain the alpha diversity results in this study. Unlike previous studies that collected samples immediately after cleaning, this study collected samples one month later, allowing dental plaque to reestablish. It has been reported that dental plaque development after cleaning exceeds initial values by the second day [74]. The observed effects of CO one month later, showing a decrease in pathogenic species and an increase in early colonizers, could indicate a return to a healthier oral microbiota.

In the present study, the effect of CO on periodontal dysbiosis was evaluated using the SMDI, which is calculated based on the median values of each genus over time for each treatment. Each genus is classified as either dysbiotic or normobiotic according to the SMDI criteria, though species-level analysis is limited by the V3–V4 sequencing technology.

The SMDI values obtained suggest that non-surgical periodontal therapy significantly reduces subgingival dysbiosis. However, the combined action of a mouthwash as an adjunct therapy, whether CHX or CO, appears to significantly reduce periodontitis-associated pathogens before and after the treatment.

CO may reduce dysbiosis predominantly through the selective inhibition of pathogenic taxa without negatively impacting beneficial bacteria. Nonetheless, future studies with additional experimental arms or more refined taxonomic methods would be valuable to further isolate and confirm the specific effects of CO on individual bacterial species within these genera. The role of inflammation in periodontitis is well-documented, with cytokines such as IL-6 and TNF-α playing central roles in disease progression. This study’s findings align with previous research that highlights the significance of these pro-inflammatory mediators in the breakdown of periodontal tissues [12]. Elevated levels of IL-6 in periodontal tissues contribute to the amplification of the inflammatory response, exacerbating tissue breakdown and bone resorption.

Similarly, TNF-α is critical in orchestrating the local inflammatory response and promoting the degradation of the extracellular matrix and bone loss through the stimulation of matrix metalloproteinases (MMPs) [13]. By enhancing the production of other inflammatory mediators, TNF-α further amplifies tissue damage, which underscores its importance as a therapeutic target [14].

The anti-inflammatory properties of CO, specifically its ability to modulate cytokine activity, suggest it may play a role in dampening the chronic inflammation seen in periodontitis. Previous studies have shown that CO’s key components, such as lauric acid, can inhibit the production of pro-inflammatory cytokines, potentially reducing the inflammatory burden in periodontal tissues [22]. This suggests that CO could mitigate the destructive effects of IL-6 and TNF-α, providing a novel approach to controlling inflammation in periodontitis.

While our findings demonstrate a significant reduction in IL-6 and TNF-α in the CO group, these two markers represent just a fraction of the inflammatory processes underlying periodontitis. Other cytokines such as IL-1β, IL-8, prostaglandin E2, and matrix metalloproteinases also play essential roles in tissue destruction and disease progression [75–77]. Although incorporating a broader panel of biomarkers would have provided deeper insights into the exact immunological pathways affected by CO, our primary objective was to evaluate two well-established mediators in periodontitis. Future research should expand on these results by examining additional inflammatory markers and exploring how they interact with the oral microbiota to influence longer-term periodontal outcomes.

Future research should investigate whether CO’s anti-inflammatory effects can effectively alter cytokine levels in clinical settings, offering a new avenue for periodontitis treatment.

## Conclusions

CO significantly reduces bacterial load in both subgingival and supragingival areas, targeting pathogenic bacteria while promoting beneficial species for a healthier oral environment. Additionally, CO reduces IL-6 and TNF-alpha levels, showcasing its anti-inflammatory properties.

These results highlight the potential clinical relevance of CO as a natural and effective adjunct in periodontal therapy, offering a promising alternative to CHX for managing periodontitis.

However, larger and long-term clinical studies are needed to assess CO’s extended effects on the oral microbiome and periodontal health. Investigating its antimicrobial and anti-inflammatory mechanisms could lead to more targeted treatments.

## Annexes I-VII

These annexes include detailed data and additional analyses.

## Electronic supplementary material

Below is the link to the electronic supplementary material.

Supplementary Material 1

## Data Availability

No datasets were generated or analysed during the current study.
