# Supplementary material for: Anti-inflammatory and antimicrobial efficacy of coconut oil for periodontal pathogens: a triple-blind randomized clinical trial
Source: Clin Oral Investig. 2025 Mar 14;29(4):182. doi: 10.1007/s00784-025-06267-8 (PMC11909057; doi:10.1007/s00784-025-06267-8)
Supplement: Supplementary file 1 — Supplementary Material 1 [file 784_2025_6267_MOESM1_ESM.pdf]

ANNEX I

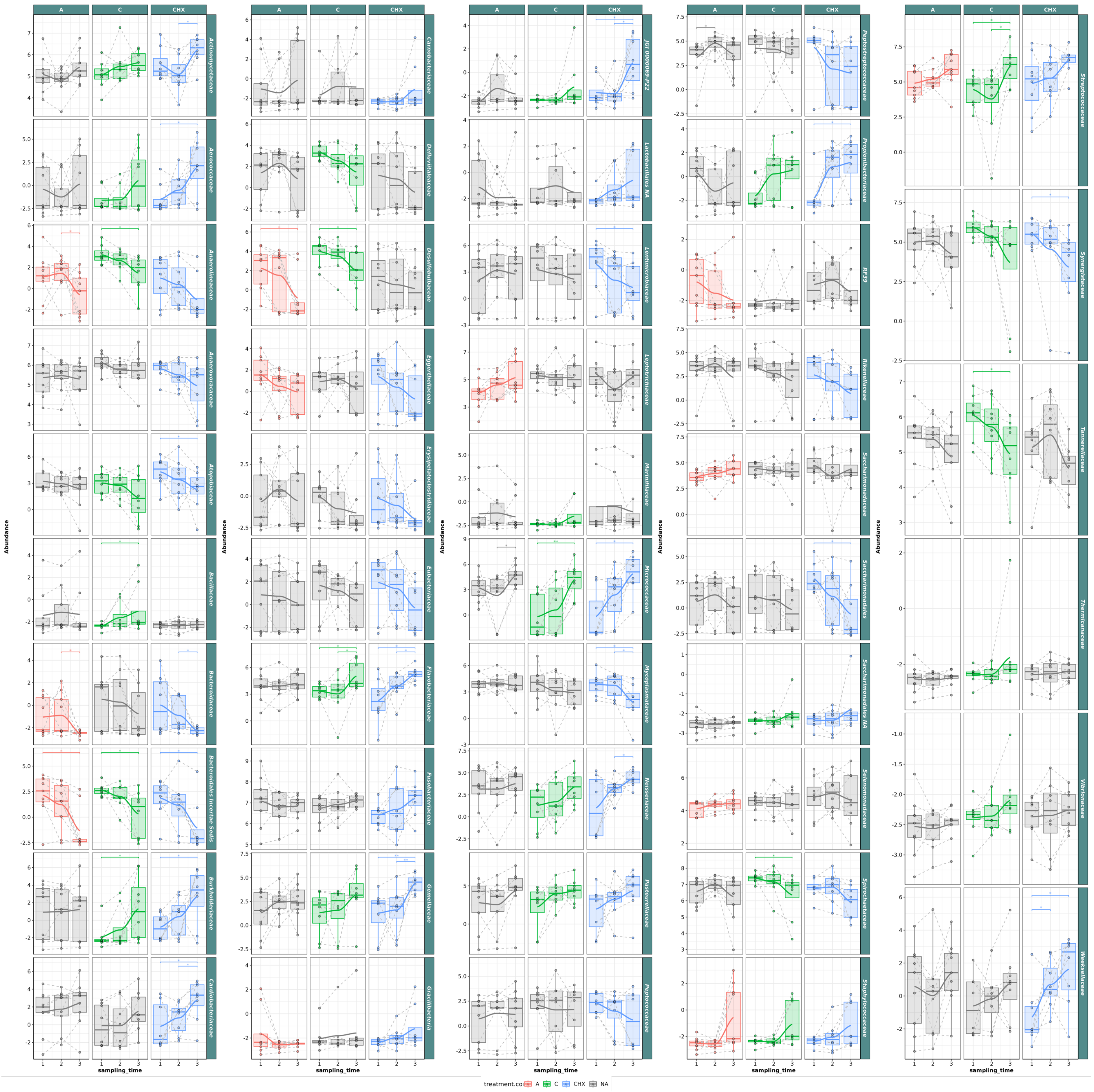

Annex I: Bacterial families of the oral microbiome of gingival crevicular fluid (GCF) samples from patients with periodontitis. TThe box plot represents the normalized Centered Log-Ratio (CLR) abundance of each family according to the type of treatment A: placebo; C: Coconut oil; CHX; and time of sampling (T1, T2 and T3). Each color belongs to a treatment in which there was a significant correlation over time, represented by a line that passes through the boxes: A) Red color: treatment with placebo, C) green color: treatment with coconut oil; and CHX) blue color, treatment with Chlorhexidine. Samples from the same patient were connected by dotted lines. The gray color is assigned when no significant correlation was obtained in the treatment. The Wilcoxon rank-sum statistical test was used: * $p < 0.05$, ** $p < 0.01$.

ANNEX II

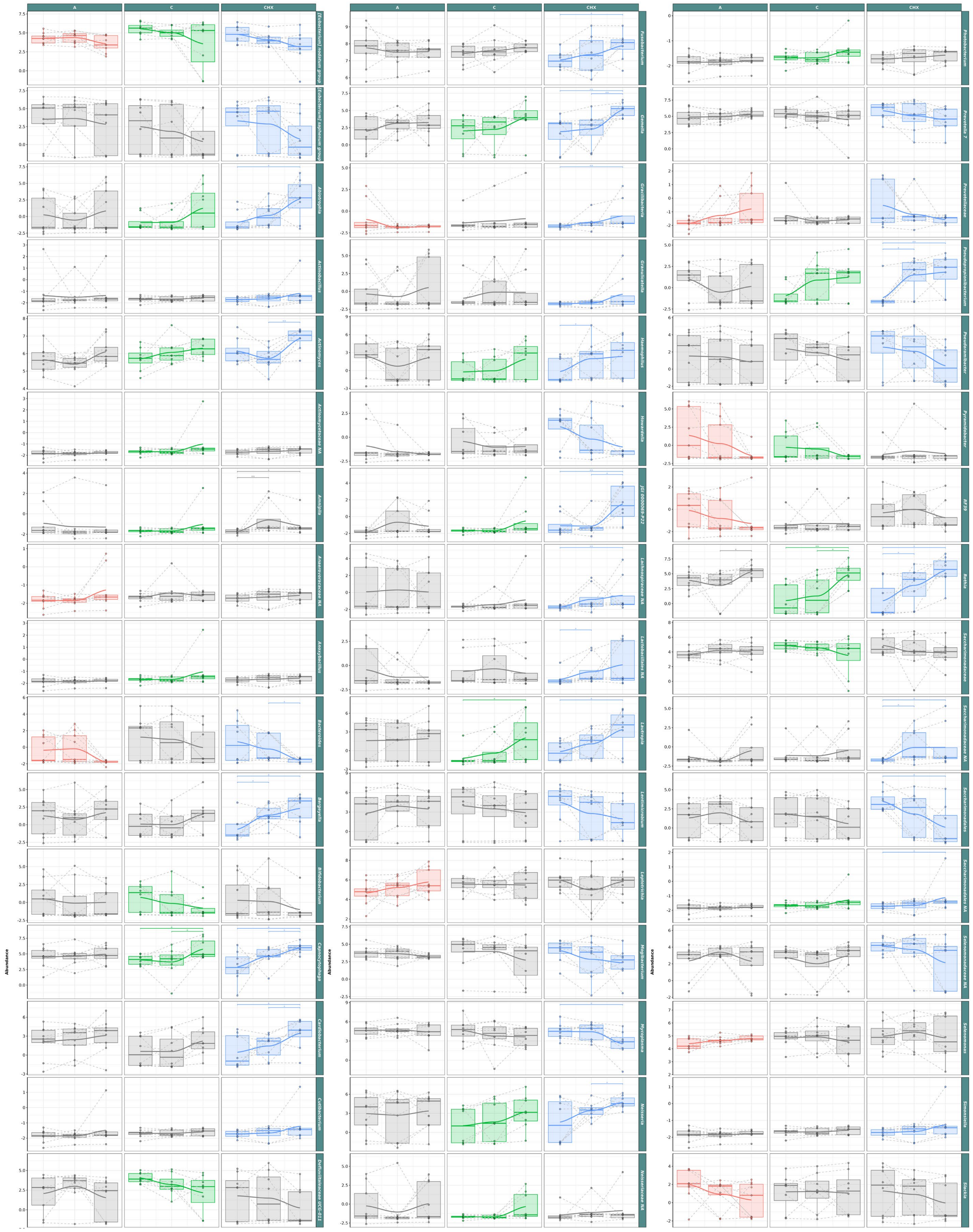

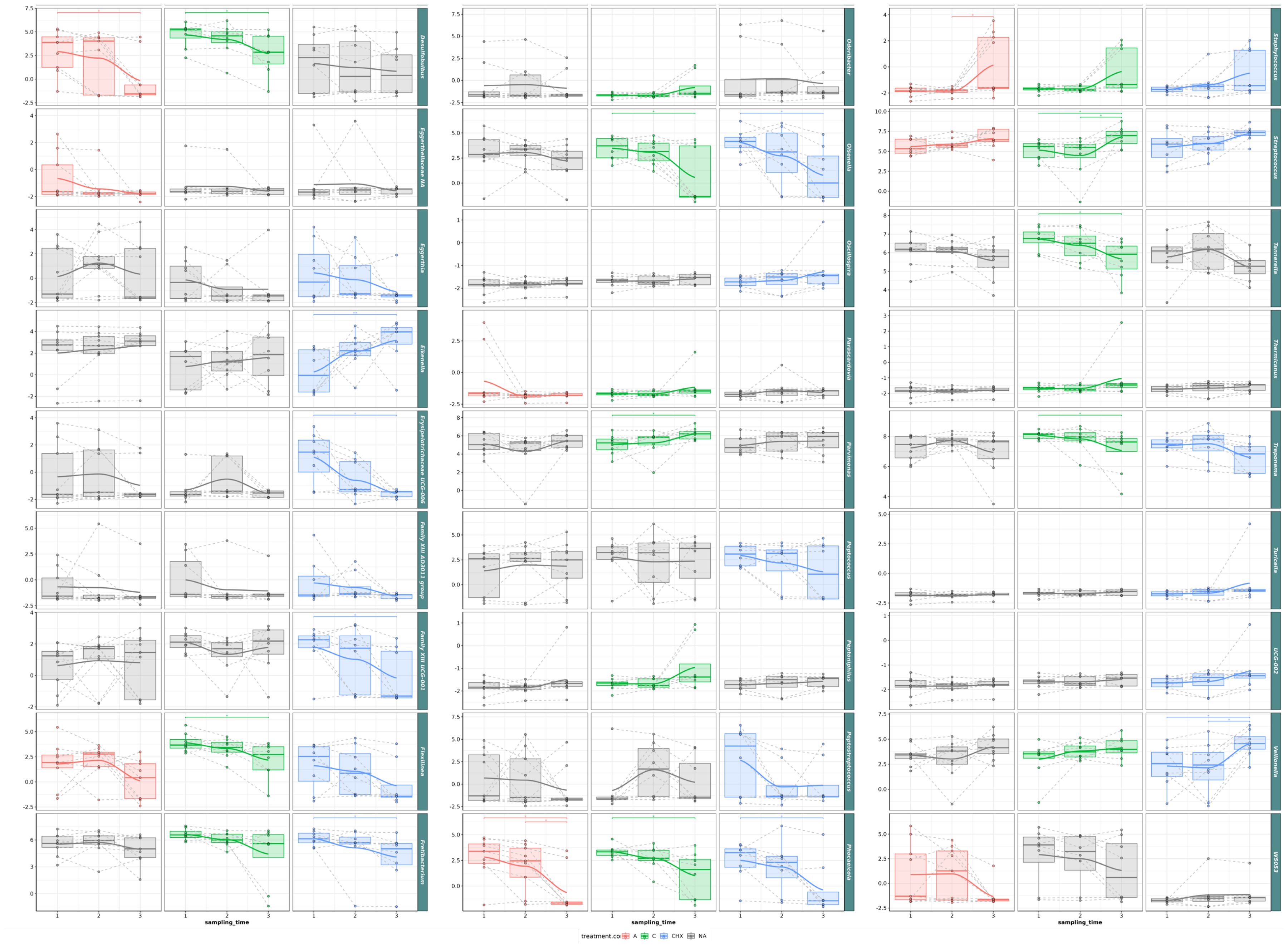

Annex II: bacterial genera present in the oral microbiome of gingival crevicular fluid (GCF) samples from patients with periodontitis. The box plot represents the Centered Log-Ratio (CLR) abundance of each genus according to the type of treatment (A: placebo; C: coconut oil; and CHX: Chlorhexidine) and time of sampling (T1, T2 and T3). Each color belongs to a treatment in which there was a significant correlation over time, represented by a line that passes through the boxes: A) Red color: treatment with placebo, C) green color: treatment with coconut oil; and CHX) blue color, treatment with Chlorhexidine. The gray color is assigned when no significant correlation was obtained in the treatment. Samples from the same patient were connected by dotted lines. The Wilcoxon rank-sum statistical test was used: * $p < 0.05$, ** $p < 0.01$.

ANNEX III

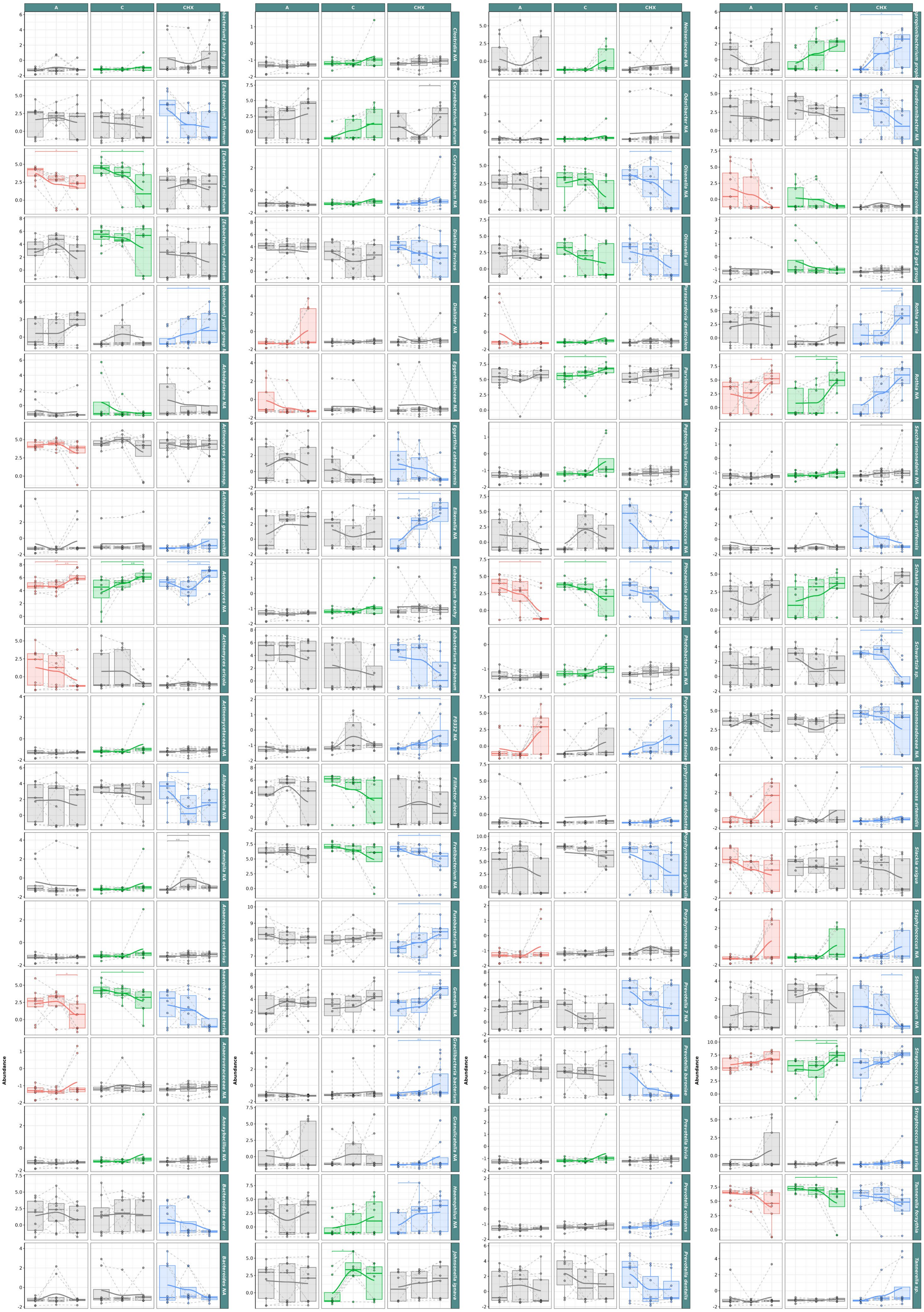
A
C
CHX
Abundance
[Eubacterium] infirmum
[Eubacterium] minutum
[Eubacterium] nodatum
Acholeplasma NA
Actinomyces NA
Actinomyces oricola
Actinomycetaceae NA
Alloprevotella NA
Anaerococcus octavius
Anaerovoracaceae NA
Anoxybacillus NA
Bacteroidales oral
Bacteroides NA
Clostridia NA
Corynebacterium durum
Corynebacterium NA
Dialister invisus
Dialister NA
Eggerthellaceae NA
Eggerthia catenaformis
Eikenella NA
Eubacterium brachy
Eubacterium saphenum
F0332 NA
Filifactor alocis
Fretibacterium NA
Fusobacterium NA
Gemella NA
Granulicatella NA
Haemophilus NA
Johnsonella ignava
Neisseriaceae NA
Olsenella NA
Olsenella uli
Parvimonas NA
Peptoniphilus lacrimalis
Peptostreptococcus NA
Phocaeicola abscessus
Photobacterium NA
Porphyromonas catoniae
Porphyromonas sp.
Prevotella 7 NA
Prevotella baroniae
Prevotella bivia
Prevotella dentalis
Pseudoramibacter NA
Rothia aeria
Rothia NA
Saccharimonadales NA
Schaalia cardiffensis
Schaalia odontolytica
Schwartzia sp.
Selenomonadaceae NA
Selenomonas artemidis
Slackia exigua
Staphylococcus NA
Stomatobaculum NA
Streptococcus NA
Streptococcus salivarius
Tannerella forsythia
Tannerella sp.

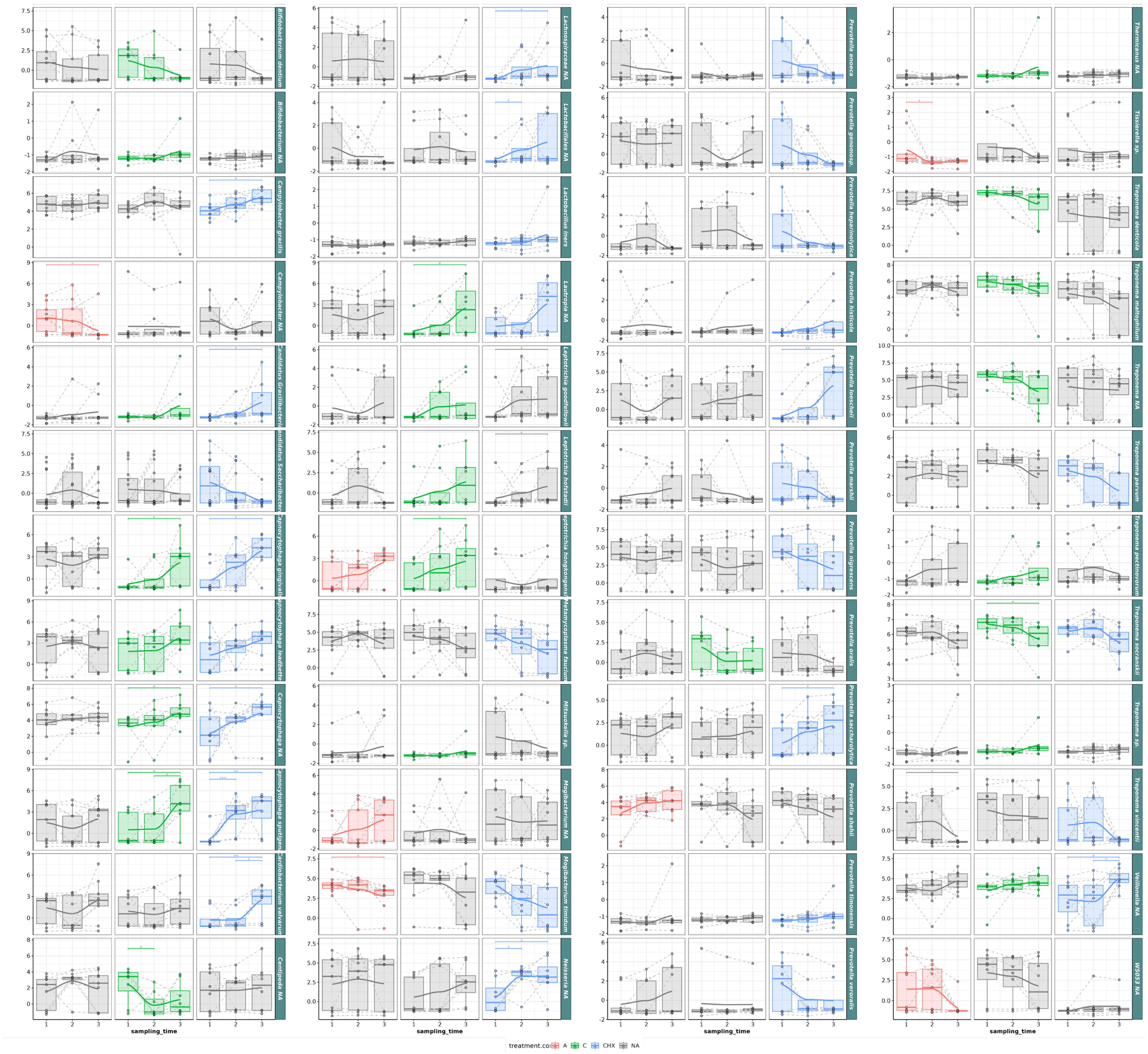

Annex III: Bacterial species present in the oral microbiome of gingival crevicular fluid (GCF) samples from patients with periodontitis. The box plot represents the Centered Log-Ratio (CLR) abundance of each species according to the type of treatment (A: placebo, C: coconut oil and CHX: chlorhexidine) and time of sampling (T1, T2 and T3). Each color belongs to a treatment in which there was a significant correlation over time, represented by a line that passes through the boxes: A) Red color: treatment with placebo, C) green color: treatment with coconut oil; and CHX) blue color, treatment with Chlorhexidine. The gray color is assigned when no significant correlation was obtained in the treatment. Samples from the same patient were connected by dotted lines. The Wilcoxon rank-sum statistical test was used: * p < 0.05, ** p < 0.01.

ANNEX IV

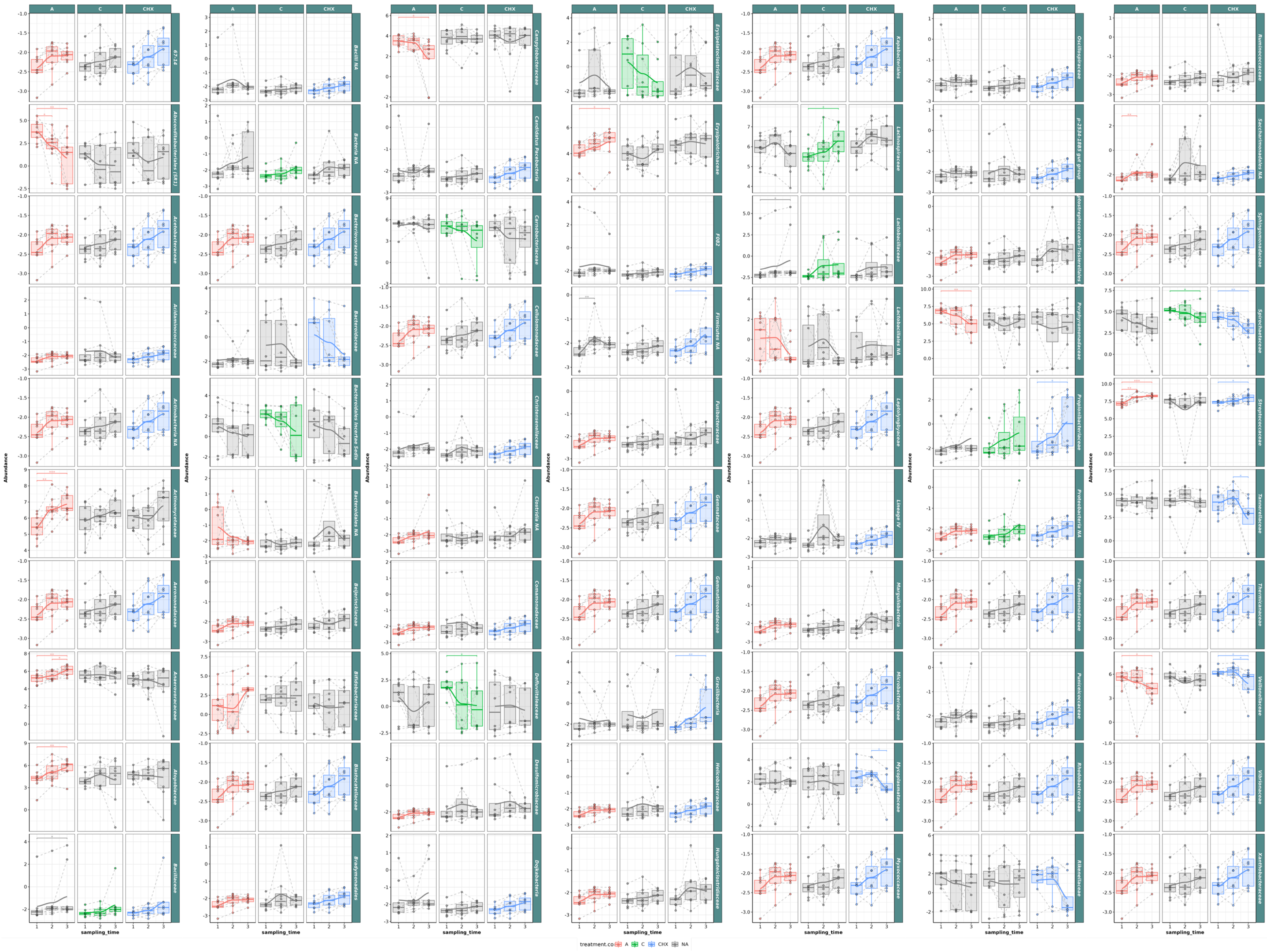

Annex IV: Bacterial families are present in the oral microbiome of saliva samples from patients with periodontitis. The box plot represents the Centered Log-Ratio (CLR) abundance of each family according to the type of treatment (A: placebo, C: coconut oil and CHX: chlorhexidine) and time of sampling (T1, T2 and T3). Each color belongs to a treatment in which there was a significant correlation over time, represented by a line that passes through the boxes: A) Red color: treatment with placebo, C) green color: treatment with coconut oil; and CHX: blue color, treatment with Chlorhexidine. The gray color is assigned when no significant correlation was obtained in the treatment. Samples from the same patient were connected by dotted lines. The Wilcoxon rank-sum statistical test was used: * $p < 0.05$, ** $p < 0.01$.

ANNEX V

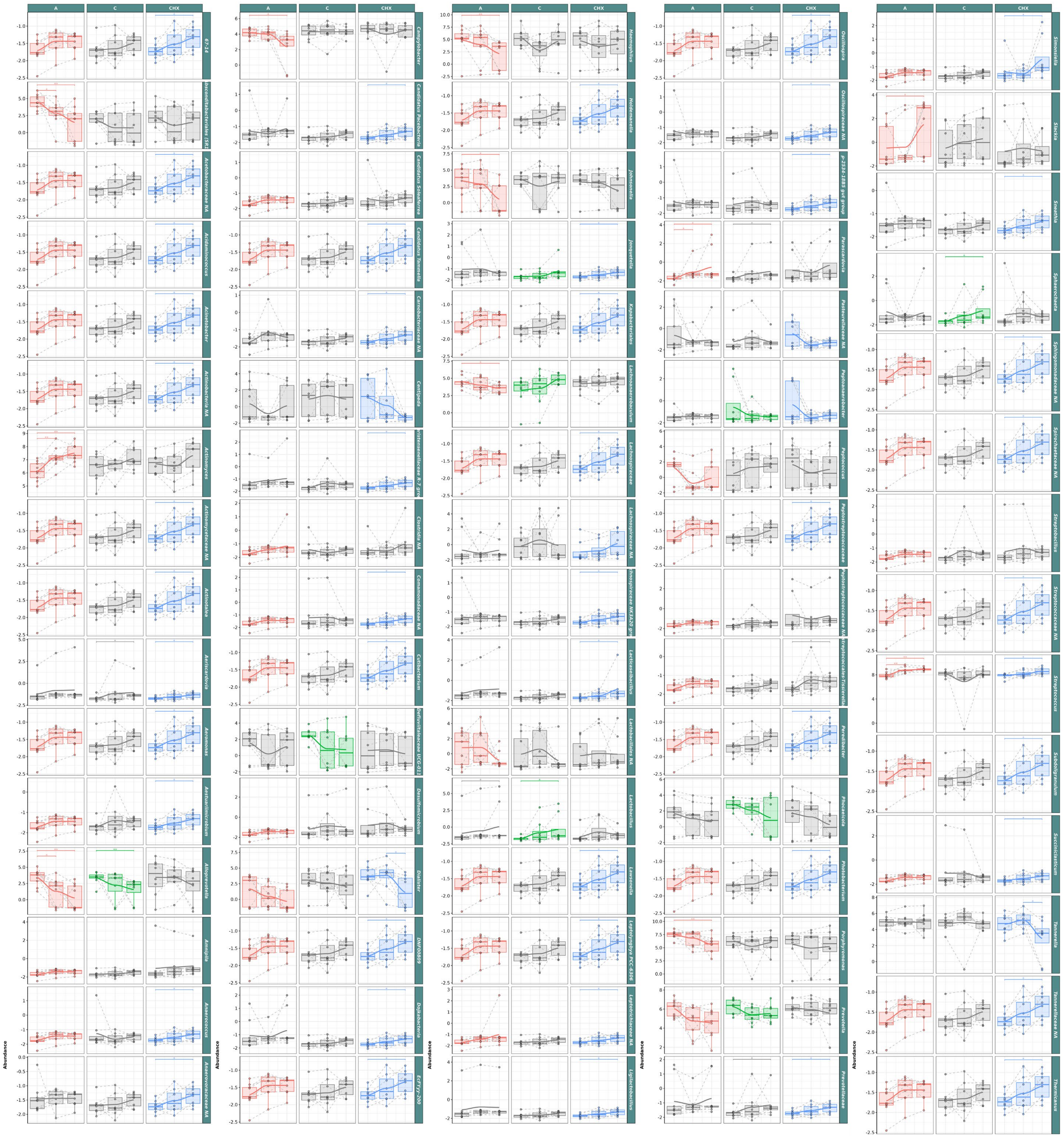

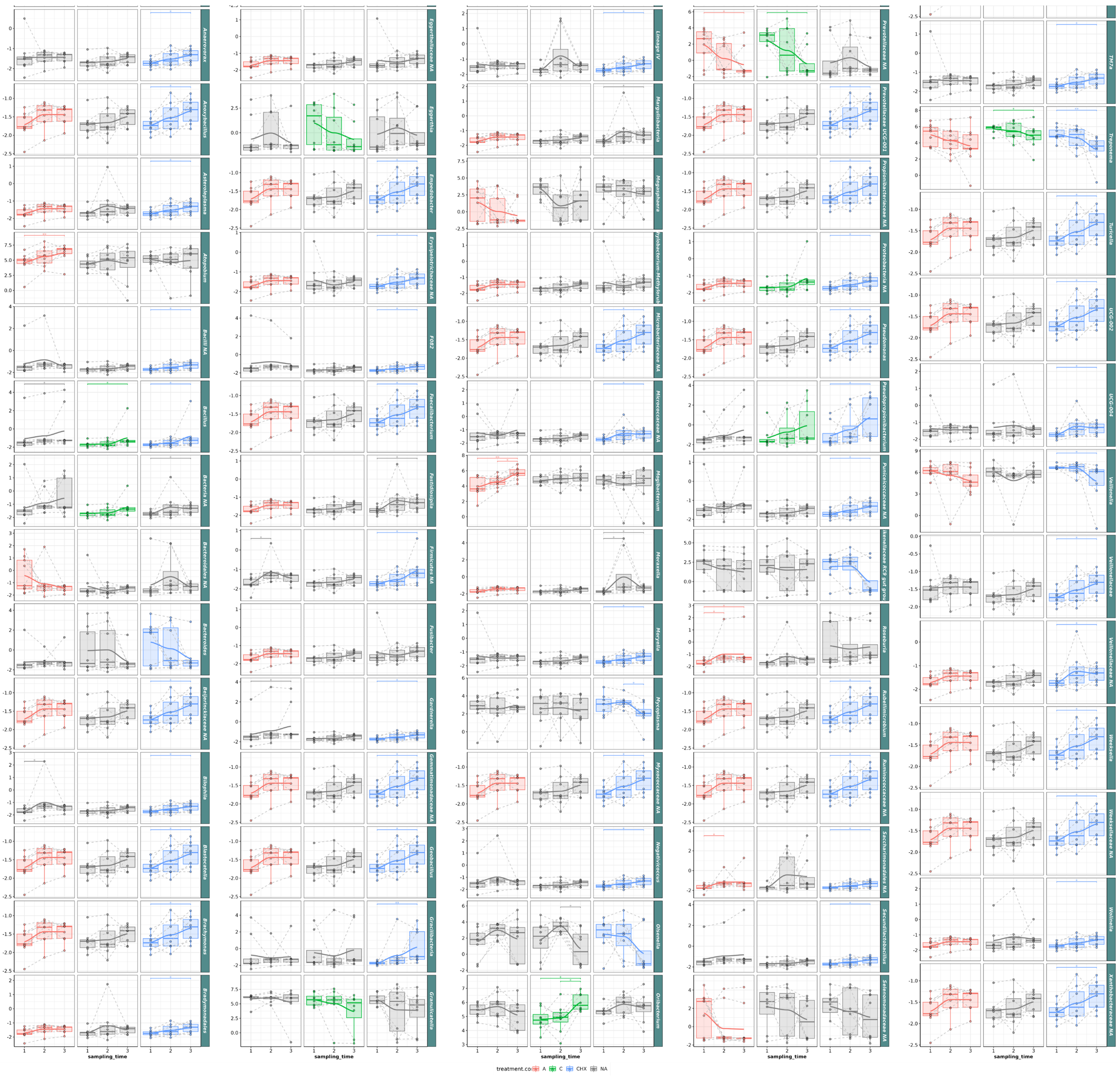

Annex V: Bacterial genera present in the oral microbiome of saliva samples from patients with periodontitis. The box plot represents the Centered Log-Ratio (CLR) abundance of each genus according to the type of treatment (A: placebo, C: coconut oil and CHX: chlorhexidine) and time of sampling (T1, T2 and T3). Each color belongs to a treatment in which there was a significant correlation over time, represented by a line that passes through the boxes: A) Red color: treatment with placebo, C) green color: treatment with coconut oil; and CHX) blue color, treatment with Chlorhexidine. The gray color is assigned when no significant correlation was obtained in the treatment. Samples from the same patient were connected by dotted lines. The Wilcoxon rank-sum statistical test was used: * $p<0.05$.

ANNEX VI

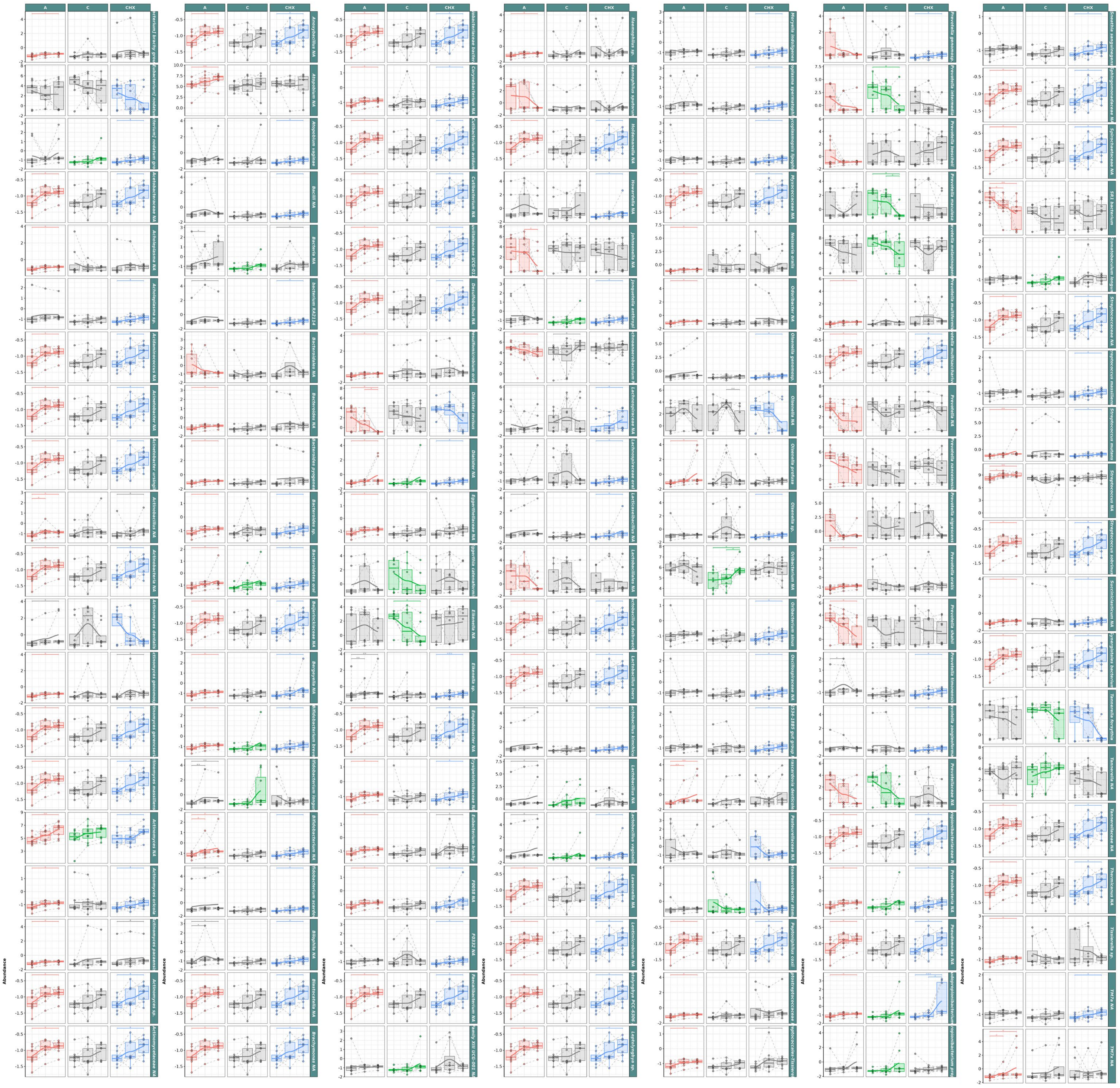

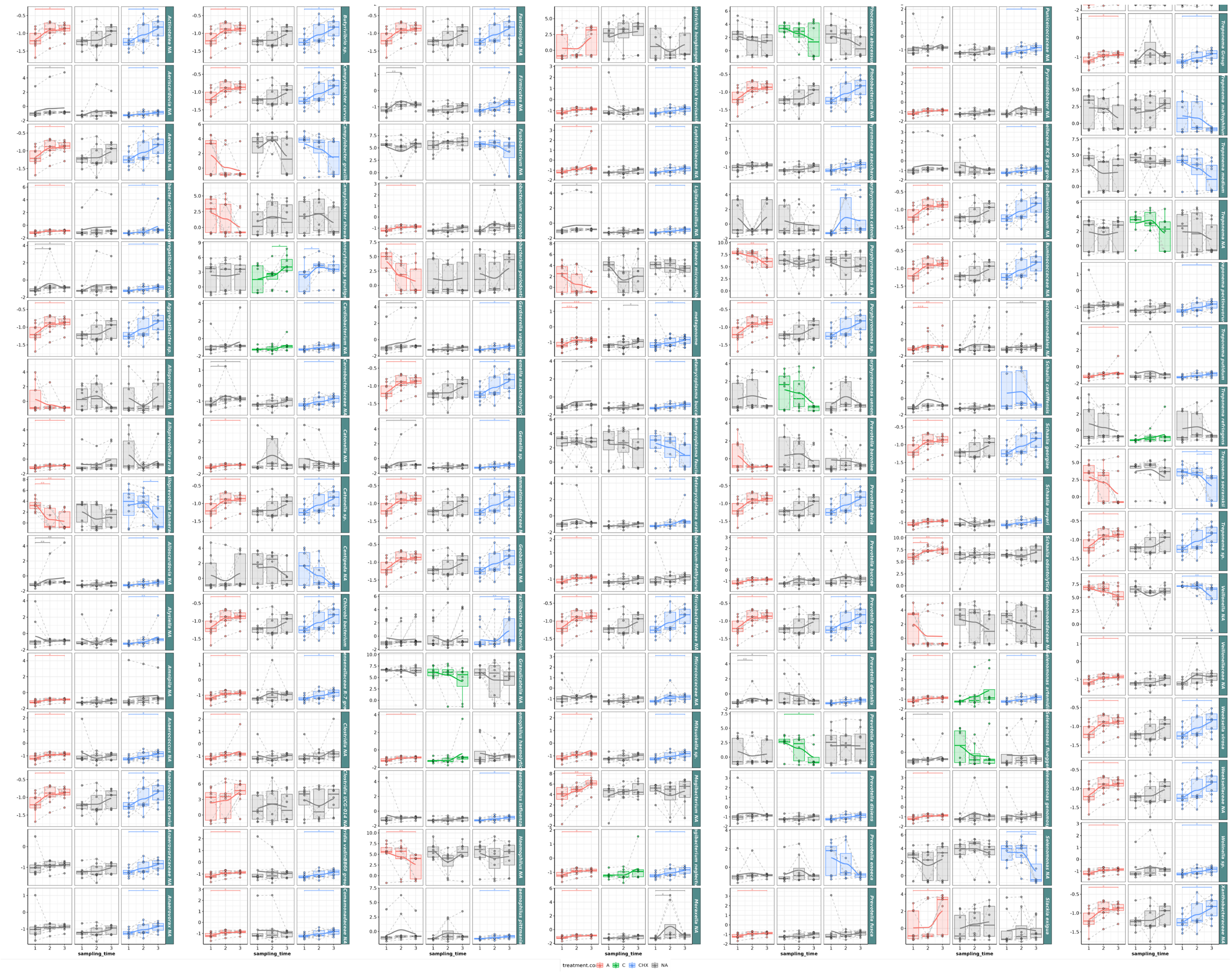

Annex VI: Bacterial species present in the oral microbiome of saliva samples from patients with periodontitis. The box plot represents the Centered Log-Ratio (CLR) abundance of each species according to the type of treatment (A: placebo, C: coconut oil and CHX: chlorhexidine) and time of sampling (T1, T2 and T3). Each color belongs to a treatment in which there was a significant correlation over time, represented by a line passing through the boxes: A) Red color: treatment with placebo, C) green color: treatment with coconut oil; and CHX) blue color, treatment with Chlorhexidine. The gray color is assigned when no significant correlation was obtained in the treatment. Samples from the same patient were connected by dotted lines. The Wilcoxon rank-sum statistical test was used: * p<0.05.

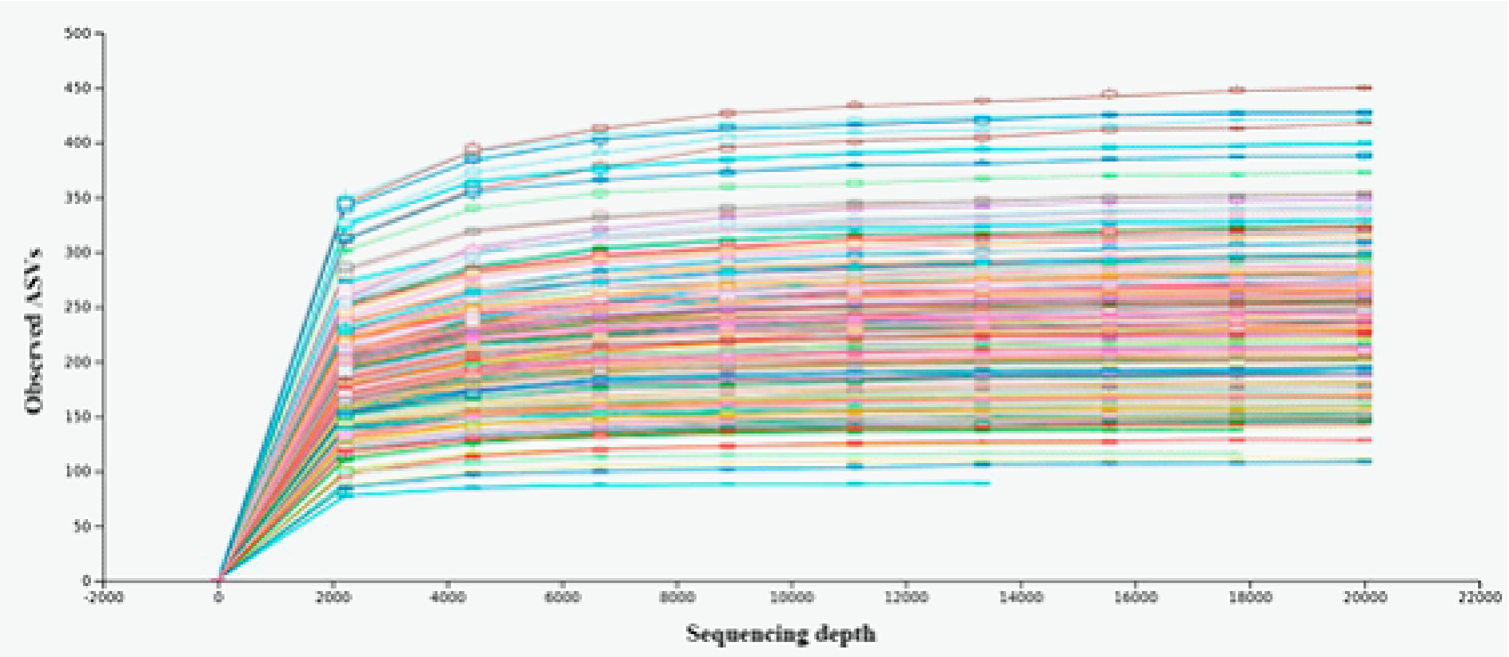

Annex VII: Rarefaction curves obtained using QIIME 2. A total of 13,223,972 reads were obtained. Following noise removal and quality control using DADA2, the number of reads decreased, improving quality. Most samples had 30,000-40,000 sequences, indicating effective filtering. Of these, 70% passed the confidence threshold, with most reads having a length of 400-450 bp, matching the expected size of the V3-V4 region of the 16S rRNA gene, confirming high-quality amplification
